# Quinoline-based Photoinitiators for Vat Photopolymerization: Synthesis and Formulation Design for 3D Printing

**DOI:** 10.1021/acspolymersau.6c00101

**Published:** 2026-08-12

**Authors:** Ilana S. Rosário, Caroline Gaglieri, Vinicius H. de Carvalho, Emily B. de Barros, João A. R. Bassetto, Gabriela Cremasco, Gilbert Bannach, Luiz C. da Silva-Filho

**Affiliations:** Faculdade de Ciências, 28108 Universidade Estadual Paulista (UNESP), Bauru 17033-260, Brazil

**Keywords:** quinoline derivatives, multicomponent reactions, radical photopolymerization, visible light curing, thioether oxidation

## Abstract

Quinoline derivatives (**Q-OMe** and **Q-SMe**) were synthesized via a green one-pot multicomponent reaction and evaluated as photoinitiators for urethane dimethacrylate (UDMA) photopolymerization. Their performance was investigated under UV (370 nm) and blue (456 nm) irradiation, in the presence and absence of ethyl *p*-dimethylaminobenzoate (EDB). Blue light led to significantly higher conversions (up to 73%) compared to UV irradiation (<5%). Statistical analysis confirmed that both the photoinitiating system and photoinitiator concentration significantly affected the final monomer conversion, with the Q-SMe/EDB system consistently exhibiting the highest conversion values. UV–vis and mass spectrometry provided evidence for the formation of a sulfoxide intermediate during Q-SMe photoactivation, supporting the proposal that this derivative can operate through both type I and type II photoinitiation pathways, whereas the photochemical behavior of Q-OMe is consistent with a predominantly type II mechanism. The optimized Q-SMe/EDB formulation (0.3 wt % Q-SMe/0.5 wt % EDB) was subsequently applied to UDMA/TEGDMA resins for vat photopolymerization, where increasing the TEGDMA content increased the penetration depth (138–172 μm) while reducing the monomer conversion. Under identical printing conditions, the optimized Q-SMe/EDB formulation produced well-defined 3D structures without overcuring, unlike the conventional CQ/EDB system. These results demonstrate the potential of quinoline-based photoinitiating systems for visible-light vat photopolymerization of methacrylate resins and highlight the importance of exploring new quinoline derivatives for the development of efficient photoinitiating systems.

## Graphical abstract

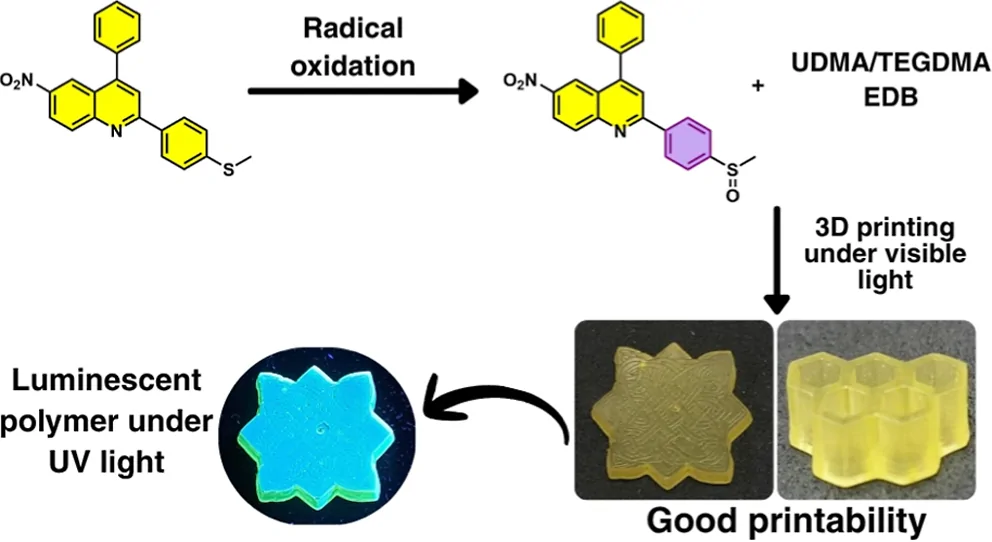

## Introduction

Plastics are a subclass of synthetic polymers that have become essential materials in modern society due to their versatility, durability, and low cost. They are widely used in everyday products such as plastic bags, toys, synthetic textiles (e.g., nylon), biodegradable sutures, dental resins, 3D printing filaments, and silicone-based medical devices like heart valves.^1,2^ In 2023, global plastic production reached approximately 413.8 million tonnes, including petroleum-based, biobased, and recycled plastics.^3^ This massive production volume and growing demand underscore the societal relevance of plastics. In this context, ongoing research into innovative and sustainable production technologies is crucial to minimizing environmental impact and advancing polymer science.

Among the various monomers available for polymer synthesis, urethane dimethacrylate (UDMA) stands out and is frequently employed in polymeric matrices and composites for biomaterial fabrication, particularly dental restoration resins. UDMA-derived polymers exhibit high mechanical strength, minimal water absorption, and excellent cross-linkability.^4–7^

Photopolymerization is a significant method for producing UDMA-derived polymers and other monomer-based materials, in which light initiates the chemical reaction between monomers.^8,9^ This technique offers ease of application and high polymerization efficiency, making it highly suitable for fields such as dentistry and 3D printing. Additionally, it exhibits lower energy consumption than thermal polymerization methods, making it a more environmentally sustainable alternative.^10,11^ The development of photopolymerization reactions has garnered increasing interest from various research groups due to their expanding range of applications and their economic, technical, and ecological advantages.^12,13^

Among emerging manufacturing technologies, additive manufacturing, particularly vat photopolymerization techniques such as stereolithography (SLA) and digital light processing (DLP), has experienced rapid growth due to its ability to fabricate complex geometries with high resolution and minimal material waste.^14,15^ In these systems, spatially controlled light irradiation enables layer-by-layer curing of liquid photopolymer resins, allowing precise control over architecture and mechanical performance. The efficiency, resolution, and mechanical integrity of 3D-printed objects are strongly dependent on the kinetics of photopolymerization, which are directly governed by the photoinitiator system.^16^ Insufficient radical generation can lead to incomplete curing, poor interlayer adhesion, and reduced mechanical strength, whereas excessive light absorption or inefficient radical utilization may limit penetration depth and printing accuracy.^17^ Therefore, the development of novel photoinitiators with optimized absorption profiles, high radical-generation efficiency, and tunable concentration-dependent behavior is essential to meet the increasing performance demands of advanced 3D printing applications.^18,19^

For photopolymerization to occur, three fundamental components are required: a monomer, a light source, and a photoinitiator (PI) or photoinitiator system (PIS).^18,20^ The PI plays a central role in the process, as it is responsible for generating reactive radicals that initiate polymerization. Radical generation occurs via two distinct mechanisms: type I and type II. In type I photoinitiation, unimolecular cleavage occurs upon light absorption, leading to the homolytic cleavage of a covalent bond, thereby forming two free radicals. In contrast, type II photoinitiation involves a hydrogen-donor compound (co-initiator) that facilitates radical generation via proton transfer, with the PI acting as a hydrogen acceptor. Commonly employed co-initiators include tertiary amines and polyalcohols.^20^ Consequently, the development of highly efficient PIs with enhanced radical-generation capacity is critical for advancing photopolymerization research. Quinoline derivatives have been widely explored in the literature due to their broad range of applications.^21,22^ Their distinctive properties, such as high thermal stability, excellent electron mobility, and strong photoluminescent efficiency, make them promising candidates for photopolymerization reactions via both type I and type II mechanisms.^9,12,23^ In this study, we report the synthesis and characterization of novel quinoline-based, evaluate their efficiency as photoinitiators, in the presence (type I) or absence of EDB (type II), and demonstrate their application in vat photopolymerization for 3D printing.

## Experimental Section

### Reagents

The reagents used in this study were used as received: 4-Nitroaniline (99%), 4-(methylthio)­benzaldehyde (95%), *p*-Anisaldehyde (∼98%), phenylacetylene (98%), urethane dimethacrylate (UDMA, ∼97%), ethyl *p*-dimethylaminobenzoate (EDB, ∼99%), ethyl acetate (∼99%), triethylene glycol dimethacrylate (TEGDMA, ∼95%), camphorquinone (CQ, 97%) acquired from Sigma-Aldrich and hydrochloric acid (HCl, 37%) from Synth.

### Quinoline Derivative Syntheses

All quinoline dyes were synthesized following the synthetic route established by Martins et al.^24^ The reaction was carried out by refluxing 1.0 mmol of *p*-nitroaniline (**1**) with 1.0 mmol of a benzaldehyde derivative (**2**) and 1.0 mmol of phenylacetylene (**3**) at 90 °C, using a 0.5 mol L^–1^ aqueous HCl solution as a catalyst for 72 h, obtaining the quinoline derivatives Q-OMe or Q-SMe as the final product. This procedure is summarized in Scheme 1.

**Scheme 1:**
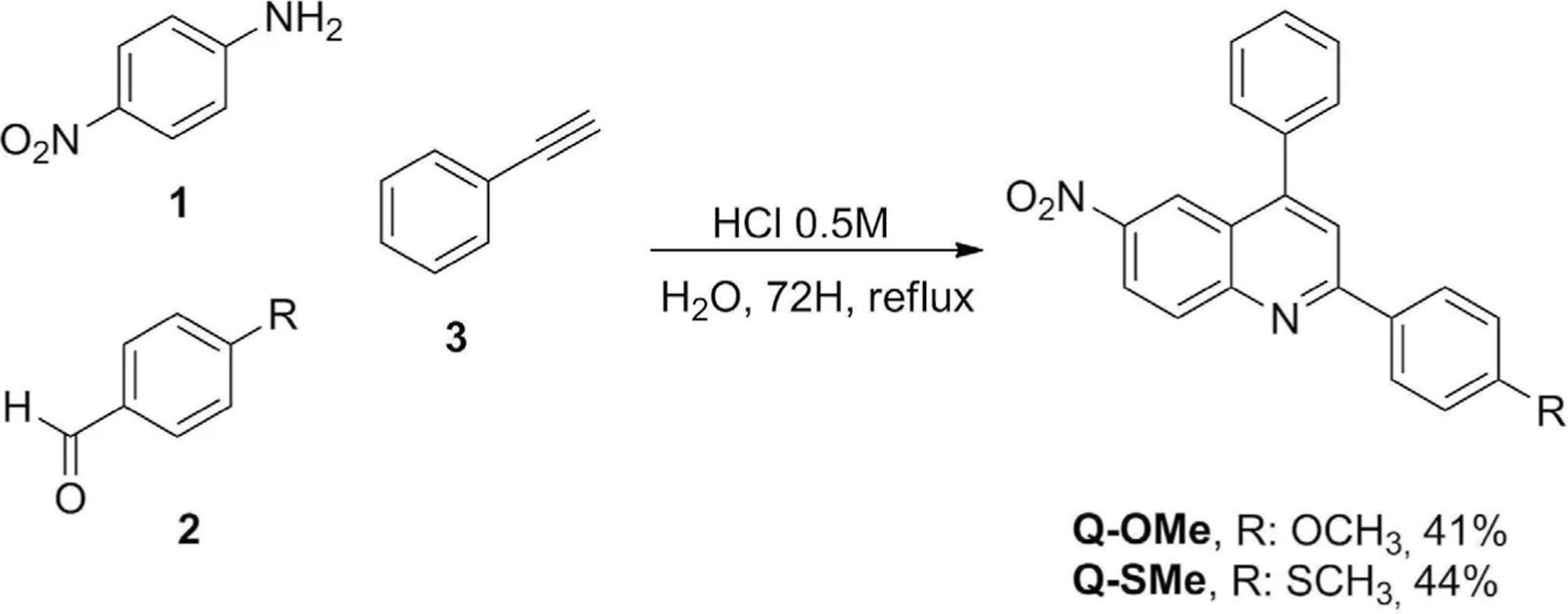
ss **Q-OMe** and **Q-SMe**

The products **Q-OMe** and **Q-SMe** were characterized by ^1^H NMR, mass spectrometry, and FTIR analyses (see Supporting Information, Figures S1–S6). Their UV–vis absorption spectra in solution and in the solid state are presented in Figure 1 and discussed below.^24,25^

**Figure 1:**
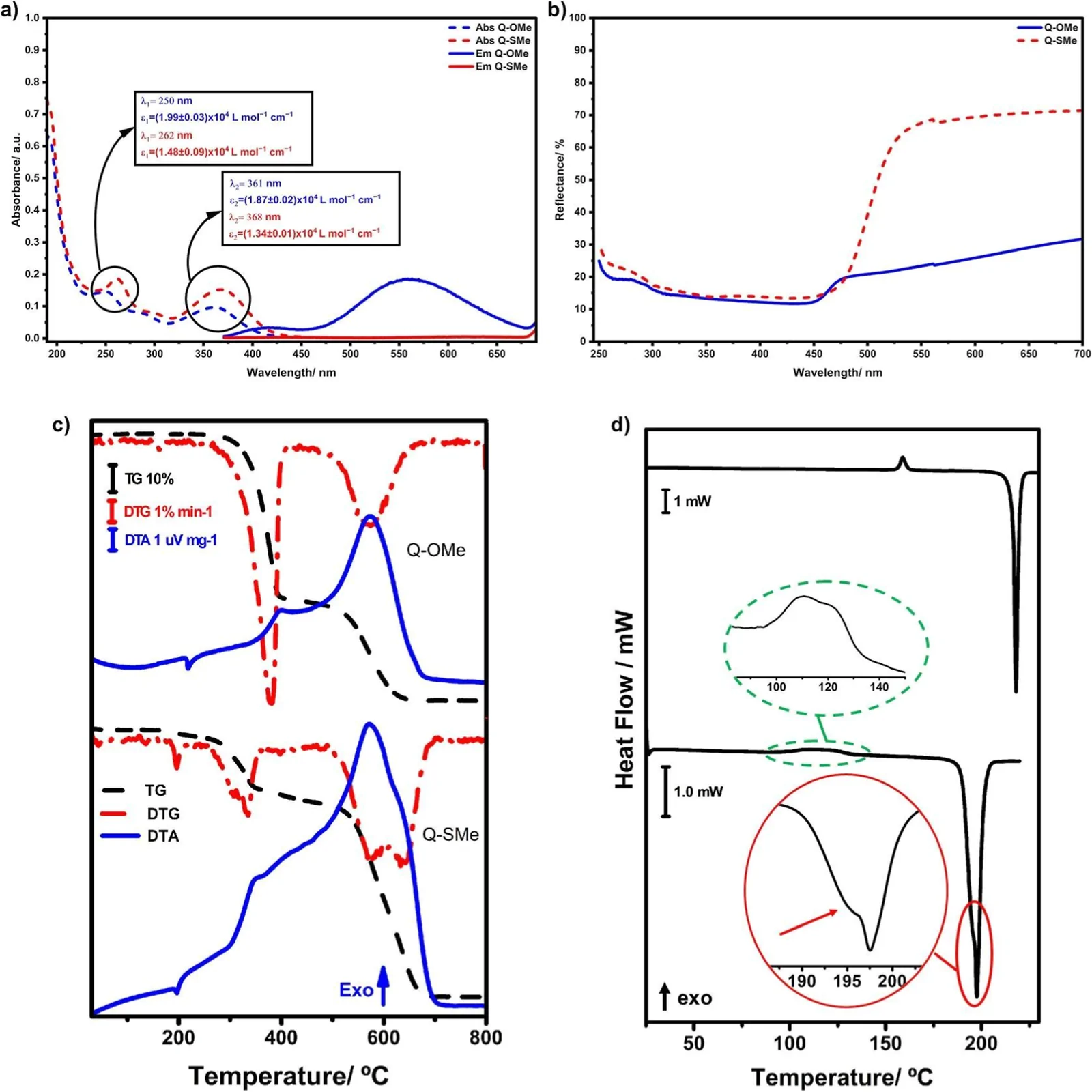
Analyses of quinoline derivatives: (a) UV–vis absorption and emission spectra in acetonitrile 5.10^–6^ M solution, (b) solid-state diffuse reflectance spectra, (c) TG/DTG-DTA curves, and (d) DSC curves (for **Q-SMe** DSC curve were obtained after heating the sample to 220 °C).

To simplify, the photoinitiator was named according to the substituent present in the quinoline derivative. Thus, the system containing the quinoline derivative substituted with a methoxy group was designated as **Q-OMe**, while the system containing the quinoline derivative substituted with a methylthio group was designated as **Q-SMe**.

### Formulations Preparation and Photopolymerization

The monomeric mixtures were prepared by adding UDMA to polypropylene containers, each containing approximately 1.0 g of monomer. The photoinitiator dye was incorporated, varying its concentration relative to the monomer mass. Ethyl *p*-dimethylaminobenzoate (EDB, 0.5 wt %) was added as a co-initiator when applicable. For the blending systems, the monomeric mixtures were prepared at a UDMA/TEGDMA mass ratio, and the amounts of initiator (0.3 wt %) and co-initiator (0.5 wt %) were based on the total monomer mass. The components were mixed until fully homogenized, and the resulting monomeric mixtures were used immediately. Table 1 presents the composition of all formulations, as well as their respective names.

**Table 1:** Composition of Each Formulation

formulation name	UDMA/wt %	TEDMA/wt %	Q-OMe amount/wt %	Q-SMe amount/wt %	CQ	EDB amount/wt %
UDMA-A1	100	0	1.0^a^			
UDMA-A2	100	0	1.0^a^			0.5^a^
UDMA-B1	100	0		1.0^a^		
UDMA-B2	100	0		1.0^a^		0.5^a^
UDMA-C1	100	0	0.7^a^			
UDMA-C2	100	0	0.7^a^			0.5^a^
UDMA-D1	100	0	0.5^a^			
UDMA-D2	100	0	0.5^a^			0.5^a^
UDMA-E1	100	0	0.3^a^			
UDMA-E2	100	0	0.3^a^			0.5^a^
UDMA-F1	100	0	0.1^a^			
UDMA-F2	100	0	0.1^a^			0.5^a^
UDMA-G1	100	0		0.7^a^		
UDMA-G2	100	0		0.7^a^		0.5^a^
UDMA-H1	100	0		0.5^a^		
UDMA-H2	100	0		0.5^a^		0.5^a^
UDMA-I1	100	0		0.3^a^		
UDMA-I2	100	0		0.3^a^		0.5^a^
UDMA-J1	100	0		0.1^a^		
UDMA-J2	100	0		0.1^a^		0.5^a^
UDMA-I2–70	70	30		0.3^b^		0.5^b^
UDMA-I2–50	50	50		0.3^b^		0.5^b^
UDMA-I2–30	30	70		0.3^b^		0.5^b^
UDMA-I2–0	0	100		0.3^b^		0.5^b^
UDMA-K2–70	70	30			0.3^b^	0.5^b^

a Based on the total mass of monomers (UDMA).

b Based on the total mass of monomers (UDMA + TEGDMA).

The initial screening was performed using two distinct light sources: an ultraviolet lamp (370 nm, Kessil KSPR160), and a blue lamp (456 nm, model KSPR160L). The systems UDMA-A1, UDMA-A2, UDMA-B1, and UDMA-B2 (Table 1) were deposited into a circular Teflon mold (diameter = 5 mm and thickness = 2 mm) and then exposed to light irradiation for 300s.

Then, the amount of photoinitiator was varied from 0.1 to 1.0 wt.% with and without a co-initiator (amount fixed at 0.5 wt %). The photopolymerization was evaluated using systems UDMA-C to UDMA-F (Table 1), under the same conditions previously described.

Finally, using the best photoinitiator system, new formulations with varying mass ratios of UDMA to TEGDMA were evaluated for vat 3D printing: UDMA-I2–70, UDMA-I2–50, UDMA-I2–30, UDMA-I2–0 (Table 1).

### Characterization and Application

#### UV–Vis Absorption Spectroscopy, Fluorescence Spectroscopy, and Electrospray Ionization Mass Spectrometry (ESI–MS)

The UV–vis spectra of quinoline derivatives were collected from solutions at 5 × 10^–6^ mol L^–1^ in acetonitrile using a spectrophotometer (Cary 8854, Agilent Technologies) with a 1 cm path length. For the solid-state analysis, spectra were collected using a PerkinElmer Lambda1050 double-beam spectrophotometer.

Molar extinction coefficients (ε_1_ and ε_2_) for the two absorption bands observed for the quinoline photoinitiators were determined from a concentration-dependent study, with absorption spectra recorded for solutions at 2 × 10^–6^, 4 × 10^–6^, 6 × 10^–6^, 8 × 10^–6^, and 10 × 10^–6^ mol L^–1^ in acetonitrile under the same instrumental conditions described above. Absorbance values for λ_max1_ (λ_max1 Q‑OMe_ = 250 nm λ_max1 Q‑SMe_ = 262 nm) and λ_max2_ (λ_max2 Q‑OMe_ = 361 nm λ_max2 Q‑SMe_ = 368 nm) were plotted against concentration, and linear regression was applied following the Beer–Lambert law (A = εlc); the slope of each fit (εl) yielded the corresponding molar extinction coefficient, since the optical path was equal to 1. The calculated ε_1_, and ε_2_ values for each photoinitiator are summarized in Table S1.

Fluorescence emission spectra were recorded using an Cary Eclipse (Agilent Technologies) spectrophotometer. The measurements were performed using the solutions prepared under the same conditions for the UV–Vis absorption experiments, consisting of the quinoline derivatives dissolved in acetonitrile at a concentration of 5 × 10^–6^ M. Emission spectra were acquired under the same experimental conditions described for the absorption measurements, using an excitation wavelength of 350 nm.

Photolysis experiments were carried out containing the quinoline derivative (Q-OMe and Q-SMe, 5.0 × 10^–6^ mol L^–1^), either alone or in combination with ethyl *p*-dimethylaminobenzoate (EDB, 5.0 × 10^–6^ mol L^–1^). The samples were irradiated at room temperature using the UV LED (370 nm) with the Kessil-PR160L as the light source, and UV–vis absorption spectra were recorded every 10 s during irradiation. The photochemical evolution of the system was monitored up to a total irradiation time of 300 s.

Mass spectra were acquired using an ion trap mass spectrometer (IT-MS, LTQ-XL, Thermo Scientific) equipped with an electrospray ionization (ESI) source and operated in positive ion mode by direct injection. The same solutions previously prepared for UV–vis analysis were employed without further modification.

#### Midinfrared Spectroscopy (MIR) and Degree of Conversion (DC %) of Monomer into Polymer

MIR spectra were collected through a diamond crystal using 32 scans and a resolution of 4 cm^–1^ in a Vertex 70 spectrometer (Bruker). The degree of conversion (DC, %) of monomers into polymers was determined based on the variation of the absorption area (A) of the C=C stretching band (∼1650 cm^–1^) associated with the UDMA structure, which is progressively consumed during the polymerization process. The analysis was performed in triplicate and in situ, by placing the monomeric mixture on the diamond window and collecting a spectrum every 10 s for 300 s. All spectra were normalized by the area of N–H stretching from the amide function (∼1530 cm^–1^) present in the UDMA chain,^26^ and the DC(%) was calculated using eq 1, in which *t* = 0 represents the total area of bands in the initial monomeric mixture and *t* = *x* corresponds to the same bands at different time points.

1 $$DC\;\left(\%\right)=\left\{1-\left[{\left(\frac{A_{1650}}{A_{1530}}\right)}_{t=x}/{\left(\frac{A_{1650}}{A_{1530}}\right)}_{t=0}\right]\right\}\times 100$$

Analysis of Variance (ANOVA) calculations and Tukey’s test were performed using Origin Pro version 8.5 (OriginLab Corporation), and Excel 2013 (Microsoft).

#### Simultaneous Thermogravimetry - Differential Thermal Analysis (TG-DTA), Differential Scanning Calorimetry (DSC), and Photo-DSC

TG-DTA curves of quinoline derivatives were obtained in STA 449 F3 equipment (Netzsch) using approximately 5 mg of sample and an open α-alumina crucible. The curves were obtained under a dry air atmosphere (70 mL min^–1^), between 30 and 700 °C, and a heating rate equal to 10 °C min^–1^. The polymers were obtained under the same conditions except for the sample mass, which was about 10 mg.

The DSC curves of quinoline derivatives and polymers were obtained using, respectively 1.5 mg and 3.0 mg of sample and acquired on the DSC1 Stare equipment (Mettler–Toledo), using a closed aluminum crucible (40 μL) with a perforated lid and a dry air atmosphere (50 mL min^–1^), and a heating rate equal to 10 °C min^–1^.

The formulations tested in 3D printing were evaluated by photo-DSC. The analyses were performed on the same equipment previously described at 25 °C (isothermal condition) and with an air flow rate of 50 mL min^–1^. Approximately 2 mg of each formulation was weighed into an open aluminum crucible (30 μL) and cured with a UV lamp Kessil KSPR160 (370 nm) for 120 s using an intensity of 100%. Two isothermal cycles were conducted, following the conditions described in the literature.^27^ Then, the second curve was subtracted from the first to obtain the total enthalpy obtained for photopolymerization (Δ*H*_total_). DC(%) for each formulation was calculated according to eq 2, in which Δ*H*_total_ is the theoretical enthalpy for a complete methacrylic double bond conversion, and d*H*/d*t* is the heat flow under an isothermal condition (*T* = 25 °C). The rate of polymerization (*R*_*p*_) was determined using eq 3, in which (d*H*/d*T)* é the heat flow, Δ*H*_total_ is the total enthalpy obtained for photopolymerization, and *t* is the time of maximum heat release (peak of DSC curve).

2 $$DC\;\left(\%\right)=\left[\frac{1}{\Delta_{total}}\times \int_{t}^{0}\left(\frac{dH}{dt}\right)T\right]\times 100$$

3 $$R_{p}=\frac{1}{\Delta_{total}}\times {\left(\frac{dH}{dt}\right)}_{T}$$

#### Working Curves and Vat 3D Printing

After determining the DC(%) (MIR and photo-DSC), as well as the thermal stability of the resulting polymers (TG/DTG), three formulations (UDMA-I2, UDMA-I2–70, and UDMA-I2–50) were evaluated using working curves, which were used to assess the curing properties following the literature.^27,28^ Critical energy (*E*_c_) and penetration depth (*D*_p_) were determined by eq 4, in which *D*_p_ is the slope, and *E*_c_ is the *x*-axis intercept. *C*_d_ is the curing depth and is determined experimentally (the thickness of one layer cured, varying the exposure time).

4 $$C_{d}=D_{p}\;\ln\left(\frac{E_{\max}}{E_{c}}\right)$$

The printing was performed in an Elegoo Mars 2 Pro DLP 3D printer equipped with a 405 nm light source (*E*_max_ = 3.8 mW cm^–2^) and an Elegoo Wash & Cure Mercury Plus for the washing and postcuring process (405 nm). The following printing parameters were adjusted using Chitubox slicing software: layer height 0.05 mm, 20 base layers, and an elevation of 5 mm after printing. The exposure times for the base layer and the remaining layers were 30 and 15 s, respectively. The postcuring period was 30 min.

## Results and Discussion

### Quinoline Derivatives Characterization

In addition to previous spectroscopic analysis, the quinoline derivatives were analyzed by UV–vis spectroscopy to improve the selection of the irradiation source.

UV–Vis spectra of **Q-OMe** and **Q-SMe** in solution are presented in Figure 1a. Both derivatives present similar spectra: an intense band centered near 225 nm, which is attributed to the π → π* transition, characteristic of conjugated aromatic systems. The second absorption region, a broader and less intense band located between 325 and 400 nm, can be associated with the n → π* transition, resulting from excitation of the nonbonding electron pairs of heteroaromatic nitrogen. The maximum absorption wavelength (λ_max_) and the molar absorption coefficient (ε) obtained for the quinoline-based molecules are provided in Table S1 (in the Supporting Information).

Upon excitation (Figure 1a), Q-OMe displayed a broad emission band centered at 562 nm, corresponding to a Stokes shift of 201 nm relative to its lowest-energy absorption band (λ_2_ = 361 nm). Such a large shift indicates substantial excited-state geometric or electronic reorganization prior to emission, consistent with charge-transfer character in the S_1_ state. In contrast, Q-SMe showed no detectable fluorescence across the entire spectral range investigated. This absence of radiative decay is attributed to the heavy-atom effect of sulfur, which enhances spin–orbit coupling and promotes efficient S_1_ → T_*n*_ intersystem crossing, effectively outcompeting the radiative pathway.^29^

Motivated by the solvatochromism previously reported by our group for quinoline derivatives,^30^ solid-state transmittance measurements were therefore performed to eliminate solvent interference and better reflect the actual curing environment (Figure 1b). Quinoline derivative compounds displayed solvent-dependent absorption profiles, with variations in band position and intensity that point to how sensitive their electronic transitions are to the local environment.^30^ Photopolymerization, however, occurs in a highly concentrated UDMA/TEGDMA resin, not in a dilute spectroscopic solvent. This distinction matters, since the absorption profile under curing conditions may differ from that measured in solution, affecting how efficiently the photoinitiator harvests incident light.

For both samples, the lowest reflectance values occurred at wavelengths below 450 nm; since the samples are opaque and do not transmit light, these minima indicate that the strongest absorption occurs in the UV and blue regions. Based on this, the photopolymerization evaluation was conducted under UV (370 nm) and blue (456 nm) light, chosen to cover the entire absorption range of the quinoline derivatives.

The TG/DTG curves of the quinoline derivatives (Figure 1c) revealed **Q-OMe** presented higher thermal stability (270 °C) compared to **Q-SMe** (180 °C). They also present different decomposition profiles, as evidenced by their TG/DTG-DTA curves (detailed discussion and table containing the mass loss details are in the Supporting Information, Table S2). The sample **Q-OMe** melts at 217.4 °C and presents elevated purity (98.8%), as evidenced by its DSC curve (Figure 1d). From the DSC curve of **Q-SMe**, it is suggested that the large endothermic event results from the melting of two distinct crystalline forms. Since this sample was previously heated in TG equipment (until 220 °C), aiming to avoid mass release inside the equipment, a qualitative study was performed to suggest that these events did not result from decomposition. A detailed discussion related to DSC analysis, as well as complementary analyses, is presented in the Supporting Information (Figures S7 and S8).

### Evaluation of Photopolymerization under Different Irradiation Sources

Systems UDMA-A1, UDMA-A2, UDMA-B1, and UDMA-B2 were submitted to two distinct light sources to verify DC (%) and *R*_p_ by MIR. Figure S9 presents the DC(%) under UV light, while Figures S10 and S11 show the representative spectra of systems under blue light, and Tables S3 and S4 present the numerical values of DC % and Rp at 10, 20, 50, 100, 200, and 300 s.

Figure 2a presents the values of DC (%), and Figure 2b presents the *R*_p_ for formulations UDMA-A1, UDMA-A2, UDMA-B1, and UDMA-B2 under blue light obtained by MIR.

**Figure 2:**
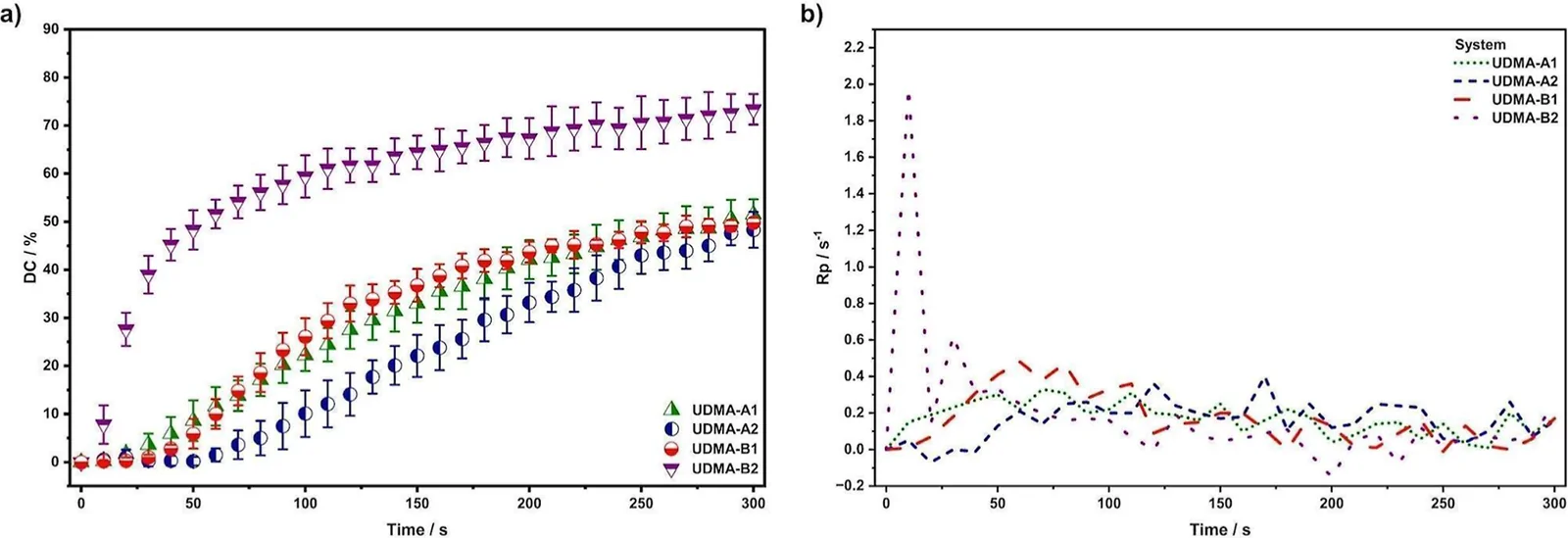
Degree of conversion of monomer into polymer [DC (%)] obtained by MIR for each formulation (a) under blue light [data are presented as mean ± standard deviation (*n* = 3)], and (b) their respective rate of polymerization (*R*_p_) under blue light.

The systems irradiated with blue light (Figure 2a) reached final conversion values of 50% or higher (at 300 s). They were higher than those obtained under UV light (Figure S9), which remained below 5% and even resulted in solid samples. Therefore, this irradiation source was chosen to perform the other tests.

Although the systems containing **Q-OMe** (UDMA-A1 and UDMA-A2) reached similar final conversions under blue light (close to 50%), UDMA-A1 exhibited higher DC(%) values throughout the polymerization process (Figure 2a). After 150 s of irradiation, it achieved approximately 33% conversion, whereas UDMA-A1 required 200 s to achieve a comparable DC(%). This behavior is further evidenced by the polymerization rate (*R*_p_, Figure 2b and Table S4), with UDMA-A1 reaching its maximum at 70 s (*R*_p_ = 0.33 s^–1^), while UDMA-A2 required 120 s (*R*_p_ = 0.36 s^–1^) to reach a comparable rate. The systems obtained with **Q-SMe** (UDMA-B1 and UDMA-B2) were more influenced by the presence of the co-initiator (EDB) than those using **Q-OMe**. In addition to the higher DC (%) values at 300 s (UDMA-B1 = 49.8 ± 1.0% and UDMA-B2 = 73.3 ± 3.4%), the maximum R_p_ was reached faster when EDB was present (UDMA-B1 at 60 s and UDMA-B2 at 10 s, 0.48 and 1.98, respectively). This influence is also reflected in the higher DC (%) values of UDMA-B2 throughout the polymerization compared to UDMA-B1, as shown in Figure 2a.

Compared with **Q-SMe**, the influence of EDB on the **Q-OMe** system was considerably less pronounced under blue-light irradiation. In addition, UDMA-B2 exhibited the highest value (73.3 ± 3.4%), which was similar to those obtained with commercial camphorquinone/EDB (∼77%) as shown in Figure S21 and in agreement with other data reported in the literature.^31^ Meanwhile, systems UDMA-A1, UDMA-A2, and UDMA-B1 presented conversion values comparable to those reported using other PIS based on quinoline derivatives for UDMA photopolymerization under blue light (44.6–54.7%).^9,12^ These findings highlight the relevance of exploring new quinoline derivatives as photoinitiators or as components of photoinitiator systems.

Q-SMe and Q-OMe responded quite differently to the addition of EDB, suggesting that the nature of the electron-donating substituent plays an important role in the photoinitiation process. Because the methylthio group (and also the sulfoxide group, which will be discussed later) is more polarizable than the methoxy group, it likely promotes a more efficient photoinduced electron-transfer process with the amine co-initiator, leading to greater radical generation. This is consistent with the superior photopolymerization performance observed for Q-SMe throughout this work, and further highlights how subtle electronic differences between substituents can significantly affect the efficiency of the photoinitiating system.^18,20^

### Influence of Photoinitiator Concentration and the Presence of a Co-initiator on the Photopolymerization

After selecting the ideal irradiation source and better understanding of the PI and PIS roles of **Q-OMe** and **Q-SMe**, the effect of their concentration in the formulation was evaluated while keeping the amount of co-initiator (EDB) constant (formulations UDMA-C1 to UDMA-J2 in Table 1).

Figure 3 displays the dependence of DC(%) on time of the different formulations irradiated by blue light, and their *R*_p_, are presented in Figure S12. Representative MIR spectra of each system are present in Figures S13–S20, in which the decrease of C=C absorption band can be clearly observed. In addition, Tables S5 and S6 provide the corresponding numerical values of DC (%) and *R*_p_ at 10, 20, 50, 100, 200, and 300 s.

**Figure 3:**
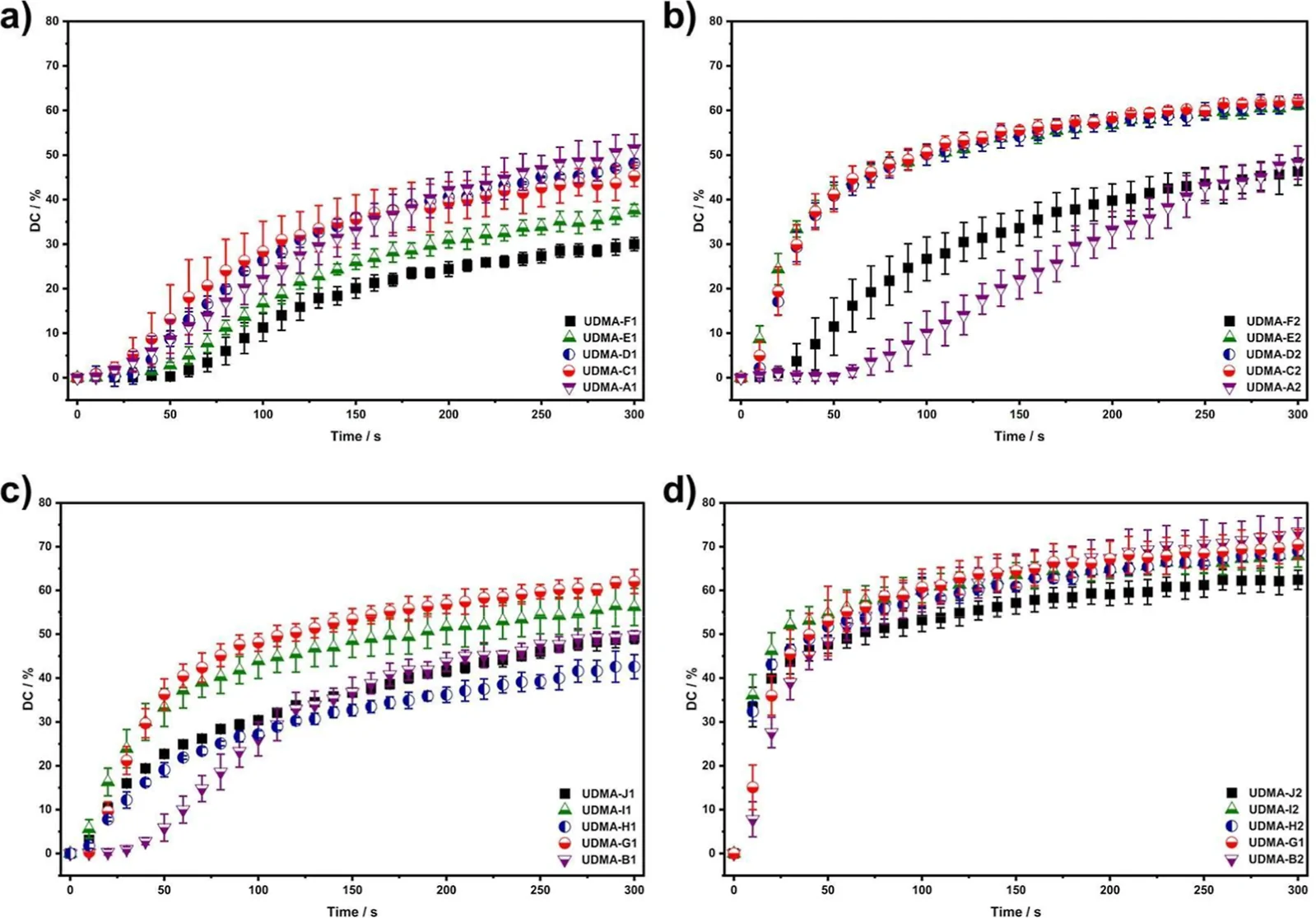
Degree of conversion of monomer into polymer [DC (%)] by MIR for formulations containing (a) **Q-OMe**, (b) **Q-OMe**/EDB, (c) **Q-SMe** and (d) **Q-SMe**/EDB at different concentrations under blue light [data are presented as mean ± standard deviation (*n* = 3)].

The pronounced gain in polymerization efficiency upon EDB addition points to a predominantly type II photoinitiation pathway for **Q-OMe**. Upon photoexcitation, the quinoline derivative is expected to interact with EDB via photoinduced electron transfer and/or hydrogen abstraction, generating the radical species that drive free-radical polymerization. This interpretation is reinforced by the photolysis experiments, which revealed no significant spectral changes for **Q-OMe** in the absence of EDB, indicating that the co-initiator is essential to the radical generation process.

Most systems containing **Q-SMe** as the PI (Figure 3c) showed final DC (%) values between 50% and 60% (Figure 3a and Table S5). In addition, the polymerization rate was higher than that observed with **Q-OMe** under the same conditions, particularly during the first 120 s. These results indicate that **Q-SMe** promotes faster radical generation, contributing to the initiation and propagation steps compared to **Q-OMe**. This behavior is highlighted in the presence of the co-initiator (EDB) (Figure 3d), where all systems exhibited DC(%) above 30% at 20 s and reached maximum *R*_p_ at 10 s (Figure 3d and Table S6).

Throughout the polymerization process, the UDMA-B1 and UDMA-B2 systems (with the maximum **tested Q-SMe** amount of 1 wt %) required more time to reach higher DC(%), and this behavior was also observed in the **Q-OMe**/EDB system (UDMA-A2). These results suggest that higher photoinitiator loading may delay the initiation and propagation steps, potentially due to light attenuation or radical recombination, although they do not necessarily compromise the final monomer conversion.

Unlike **Q-OMe**, then, **Q-SMe** appears able to promote efficient radical generation even without EDB—a finding consistent with its photolysis behavior. The significant improvement seen after EDB addition, however, shows that a Type II pathway still contributes to initiation. Q-SMe therefore seems to combine direct radical generation with a co-initiator-assisted mechanism.

A two-way ANOVA of final DC % values indicated a significant effect of both the system and concentration factors, as well as a significant interaction between them (*p* < 0.05), with differences between means identified by Tukey’s test (lowercase letters: concentrations within the same system; uppercase letters: systems at the same concentration).

The Q-OMe system was the only one to exhibit a dose–response behavior consistent with classical expectations: an increase in DC with increased significantly and progressively from 0.1% to 0.3%, reaching its highest value at 0.5%; the values at 0.7% and 1% did not differ statistically from 0.5%, establishing a higher plateau from that concentration onward. The Q-OMe + EDB and SMe systems showed nonmonotonic behavior, with an initial increase in DC followed by a significant decrease at higher concentrations: in the Q-OMe + EDB system, there was a significant increase from 0.1% to 0.3%, with the values at 0.5% and 0.7% remaining statistically similar; at 1%, a significant reduction was observed, returning to the statistical level of the lowest concentration, and in the Q-SMe system, final DC also increased from 0.1% to 0.3%, followed by a significant decrease at 0.5% and a further increase at 0.7%; at 1%, the value returned to a level statistically equivalent to that of 0.1%.

The Q-SMe + EDB system stood out for presenting the highest absolute final DC % values at all concentrations and for maintaining a broad statistical plateau (0.3% to 0.7%), with an additional increase only at the highest concentration (1%), which nonetheless did not differ statistically from the intermediate concentrations. This behavior indicates greater robustness and tolerance of this system to variations in agent concentration, suggesting a more favorable chemical compatibility between the B2 matrix and the additive, resulting in higher conversion that is less sensitive to formulation fluctuations, a desirable characteristic from a reproducibility standpoint.

Regarding the comparison between systems at each concentration, Q-SMe + EDB showed the highest final DC values at all concentrations tested, while Q-OMe consistently showed the lowest values at most concentrations.

These findings highlight the importance of systematically evaluating photoinitiator concentration when developing new photoinitiating systems. Formulation optimization should weigh both the initial polymerization kinetics and the final monomer conversion, since higher photoinitiator loading does not necessarily translate into better photopolymerization performance.^32–35^

### Photolysis in the Presence and Absence of Co-Initiator

To evaluate possible changes in the photochemical behavior of the quinoline derivatives under irradiation, as well as the influence of the co-initiator on their light-induced reactivity, a photolysis study was performed. The spectra obtained under UV and blue lights are presented in Figures 5 and S22, respectively.

**Figure 4:**
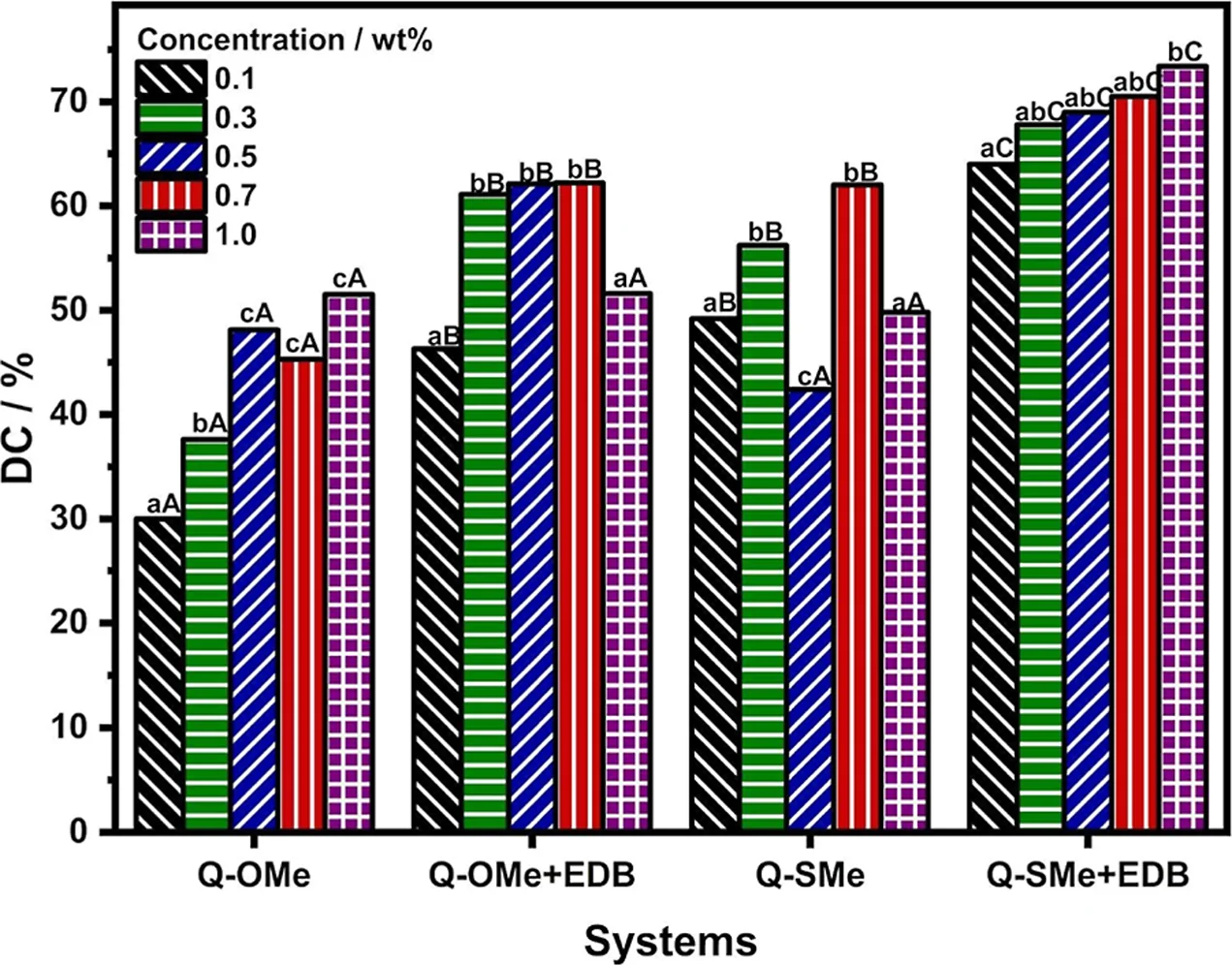
Degree of conversion of monomer into polymer (DC(%)) by MIR at different concentrations under blue light (Different lowercase letters indicate significant differences among concentrations within the same resin system, while different uppercase letters indicate significant differences among resin systems at the same concentration (two-way ANOVA followed by Tukey’s post hoc test, *p* < 0.05)).

**Figure 5:**
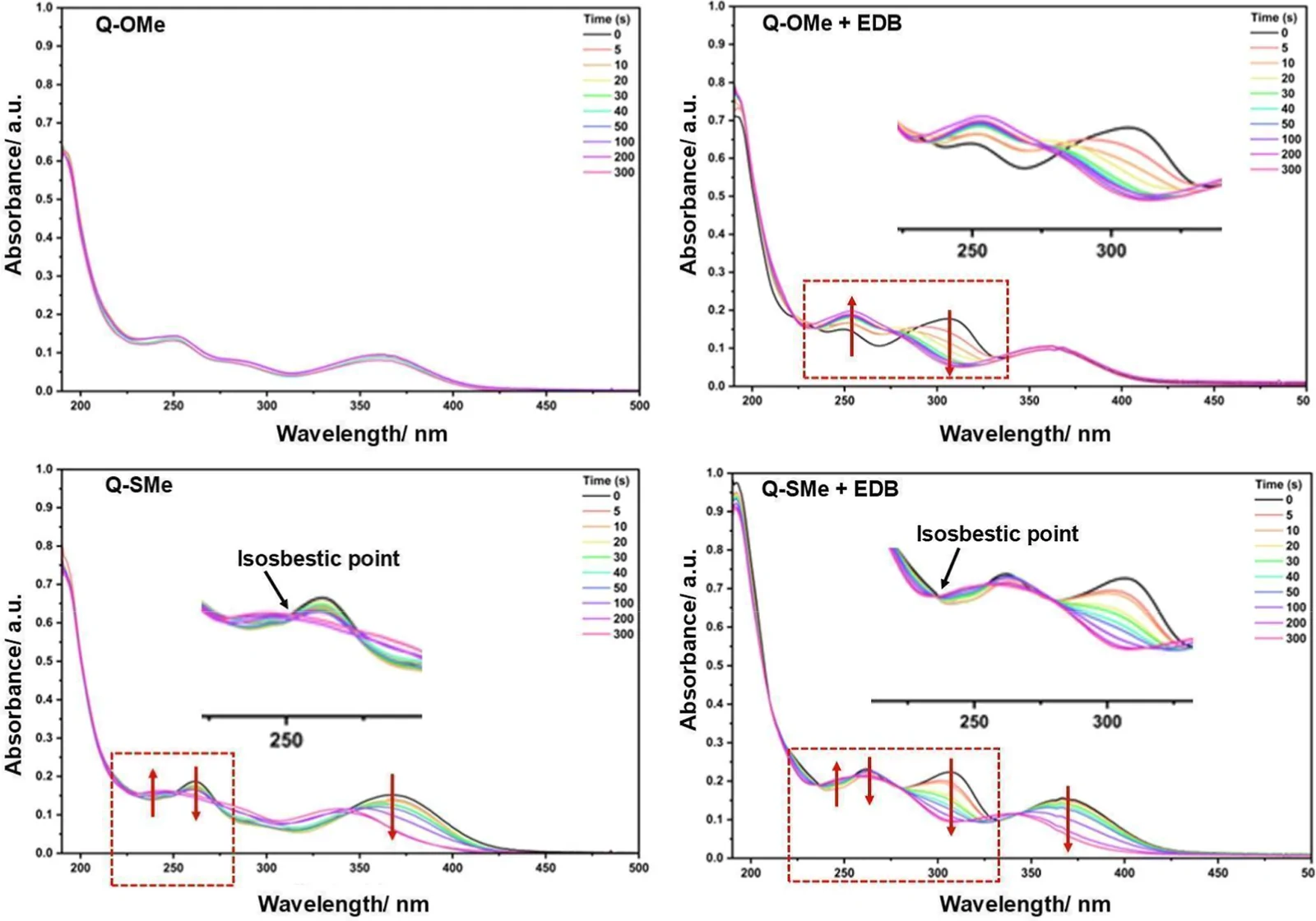
UV–vis spectra obtained for systems with **Q-OMe** and **Q-SMe**, in the absence and presence of EDB, after different times under UV irradiation.

Quinoline derivatives presented different behaviors under the photolysis experiment. In the absence of EDB, no significant changes are observed in the **Q-OMe** spectra. The initial spectrum of the system containing EDB (black line in **Q-OMe**+**EDB**) presents an additional band compared to the system without it. The band is centered at 310 nm and can be associated with the absorption of EDB, which was confirmed by the UV–vis spectrum of EDB in acetonitrile (Figure S23). As observed in the figure, this band decreased over time, suggesting that it is proportional to the hydrogen abstraction of EDB in the photoinitiation process. Meanwhile, the band centered at 255 nm increases, which may result from a new species of EDB or from the phenolic structure resulting from the quinoline rearrangement. This new structure was previously reported by our group for other quinoline derivatives with a nitro substituent.^9^

Although the quinoline derivative **Q-SMe** also presents a nitro substituent in its structure (Scheme 1), based on the UV–vis spectra resulting from photolysis, it seems that it did not follow the same initiation mechanism, since significant changes are observed throughout the irradiation exposure, even without EDB. The bands centered at 370 and 260 nm clearly decreased over time, while the absorption centered at 240 nm increased. An isosbestic point at 253 nm is also observed, indicating the equilibrium between two distinct species in the system without EDB (**Q-SMe**). A similar behavior is observed in the presence of EDB (**Q-SMe**+**EDB**), in which, besides the decrease of the bands centered at 370 and 253 nm and the increase of that centered at 240 nm, the reduction of the EDB band (centered at 310 nm) is also observed. These results suggest that the quinoline derivative **Q-SMe** can act as a type I photoinitiator, being able to generate a radical without the presence of a co-initiator.

The performed photolysis tests were qualitative and do not necessarily correlate with photopolymerization efficiency. They show the consumption and transformation of the photoinitiator upon irradiation, whereas polymerization efficiency depends on the generation of reactive radicals capable of initiating and propagating polymer chains. A higher degree of photolysis, then, may simply reflect greater photochemical conversion of the initiator, without implying that the resulting photoproducts effectively contribute to radical-mediated polymerization. This helps explain why more extensive photolysis is observed under UV irradiation, while higher polymerization efficiencies occur under blue-light irradiation, a contrast showing that productive radical generation, rather than photoinitiator consumption, ultimately governs polymerization performance. In addition, the photopolymerization role also depends on the diffusion of radicals and light in the monomeric mixture, which will be different from that observed in diluted liquid solutions using solvents (acetonitrile in the present work).

The mechanism of the **Q-OMe** photoinitiator should follow a similar pathway (Type II) for other quinoline derivatives described in a previous paper.^9^

Moreover, based on the results presented here, Q-SMe may act as a Type I and Type II photoinitiator. A mechanism of **Q-SMe** oxidation and a possible way for its photoinitiator role (in the absence of EDB) is proposed in Figure 6a. Upon light irradiation (Figure 6a), the Q-SMe chromophore is excited from its ground state (S_0_) to the first singlet excited state (S_1_), and subsequently undergoes intersystem crossing (ISC) to the triplet excited state (T_1_). This triplet state is proposed to transfer its excitation energy to ground-state molecular oxygen (^3^O_2_), generating singlet oxygen (^1^O_2_).^36,37^ The resulting ^1^O_2_ is then proposed to react with a ground-state Q-SMe molecule through a secondary oxidation step, occurring preferentially at the sulfur center and initiating the oxidation pathway described below. Such sulfur oxidation behavior is well documented in the literature for thioether compounds.^36^ This unstable species subsequently undergoes rearrangement and bond reorganization, leading to oxygen transfer at the sulfur center and formation of the corresponding sulfoxide (**Q-Sulfoxide**). Subsequently, as depicted in Figure 6b, a photochemical pathway involving homolytic cleavage at the sulfur–methyl bond of the oxidized quinoline is proposed, leading to the formation of radical species. The oxidation process under irradiation and the possible radical generation are already described in the literature for other **SMe** molecules.^36,37^

**Figure 6:**
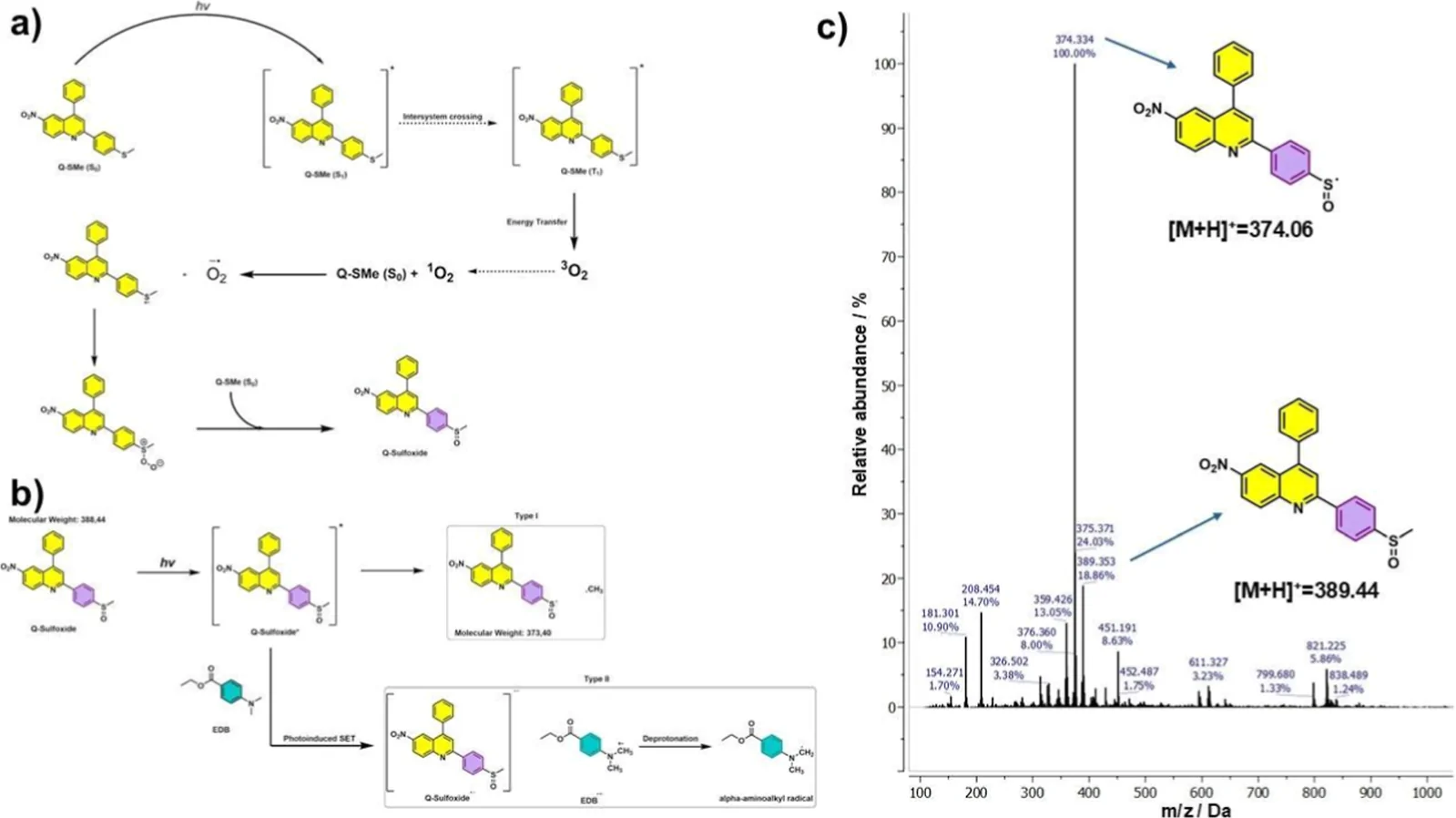
(a) Proposed oxidation pathway of the quinoline structure by atmospheric oxygen. (b) Suggested homolytic cleavage mechanism. (c) Mass spectrum highlighting the molecular ion at *m*/*z* 389.06, attributed to the oxidized quinoline species.

The self-sensitized photooxidation of aryl sulfides to the corresponding sulfoxides via singlet oxygen, proceeding through a persulfoxide intermediate, is a well-established mechanism in the literature.^38–40^ To evaluate if this suggestion could be used here, the final solutions from the Q-SMe photolysis were analyzed by direct-injection mass spectrometry, and the resulting spectrum is shown in Figure 6c. It confirms the formation of the Q-sulfoxide species: the peaks at *m*/*z* 374.06 and 389.44 ([M + H]^+^) correspond to the type I cleavage product and the intact Q-Sulfoxide photoproduct, respectively, consistent with the mechanism proposed in Figure 6a. Interestingly, the mass spectra obtained from Q-SMe photolysis in the presence and absence of EDB were essentially identical (Figure S24), so no stable adduct or covalently modified product involving EDB could be detected under these conditions. UV–vis monitoring of the photolysis (Figure 4), however, complements these results: the absorption band attributed to EDB decreased progressively throughout irradiation, confirming that the co-initiator is indeed consumed during the process. This consumption is consistent with EDB acting as the electron/hydrogen donor in the type II mechanism (Figure 6b), first oxidized to its radical cation, then converted into the aminoalkyl radical (EDB­(-H)^•^) responsible for initiating polymerization. No discrete EDB-derived species could be identified by mass spectrometry, likely owing to the instability and high reactivity of this radical intermediate.

Therefore, these results indicate that molecular oxygen plays an active and beneficial role in the activation of the Q-SMe photoinitiator, being consumed in situ to generate the photoactive Q-Sulfoxide species prior to radical formation. It is important to emphasize that the present work does not evaluate oxygen inhibition during polymerization; rather, it provides evidence that oxygen participates in the activation pathway of the Q-SMe photoinitiator by promoting the photooxidation of the methylthio substituent, leading to sulfoxide formation before radical generation. This oxidation step is distinct from the subsequent polymerization process, during which molecular oxygen can still decrease the rate of free-radical polymerization. This effect was not evaluated, since the experiments were performed under ambient atmosphere to reproduce the conditions employed during vat photopolymerization.

These results indicate that the **Q-SMe** quinoline derivative exhibited greater potential as a photoinitiator for 3D printing compared to **Q-OMe**. In addition, its performance is significantly enhanced when used as part of a photoinitiating system in the presence of EDB. Based on these results, the UDMA-I2 formulation was selected for the subsequent experiments. This formulation contains 0.3 wt % **Q-SMe** and 0.5 wt % co-initiator (EDB).

### Evaluation of Formulations Containing TEGDMA for VAT Polymerization 3D Printing

After evaluating the irradiation source, the PIS system, and its concentration, we evaluated the system in VAT photopolymerization 3D printing. It is well-known that UDMA exhibits high viscosity for this process, and its printability can be improved by using a reactive or nonreactive diluent or mixing it with other monomers. Therefore, aiming to assess the suitability of the PIS for 3D printing, we chose TEGDMA, which is a well-known low-viscosity dimethacrylate monomer that has been widely employed in photopolymerizable systems with UDMA and other dimethacrylate monomers in VAT photopolymerization or not. The formulations varied UDMA/TEGDMA mass proportions, keeping the concentration of Q-SMe and EDB constant (Table 1, systems UDMA-I2–70, UDMA-I2–50, UDMA-I2–30, and UDMA-I2–0).

Previously to the printing tests, the formulation had the DC (%) and *R*_p_ (s^–1^) determined by photo-DSC (eqs 2 and 3). The resulting curves and polymers are present in Figure 7a–c, while the numerical results are presented in Table 2. As UDMA decreases in formulations, the heat released by the reaction also decreases, as evidenced by the total enthalpy variation (Δ*H*_*T*_). Although all systems present peak temperatures between 5 and 7 s, the values of maximum rate of polymerization (*R*_pmax_) varied considerably, with the systems UDMA-I2–30 and UDMA-I2–0 presenting the lowest values. In addition, after 120 s of light exposure, these two systems did not achieve the gel point, remaining liquid at the end of the analysis (Figure 7d). This is supported by their lower DC (%) (<30%, Table 2).

**Figure 7:**
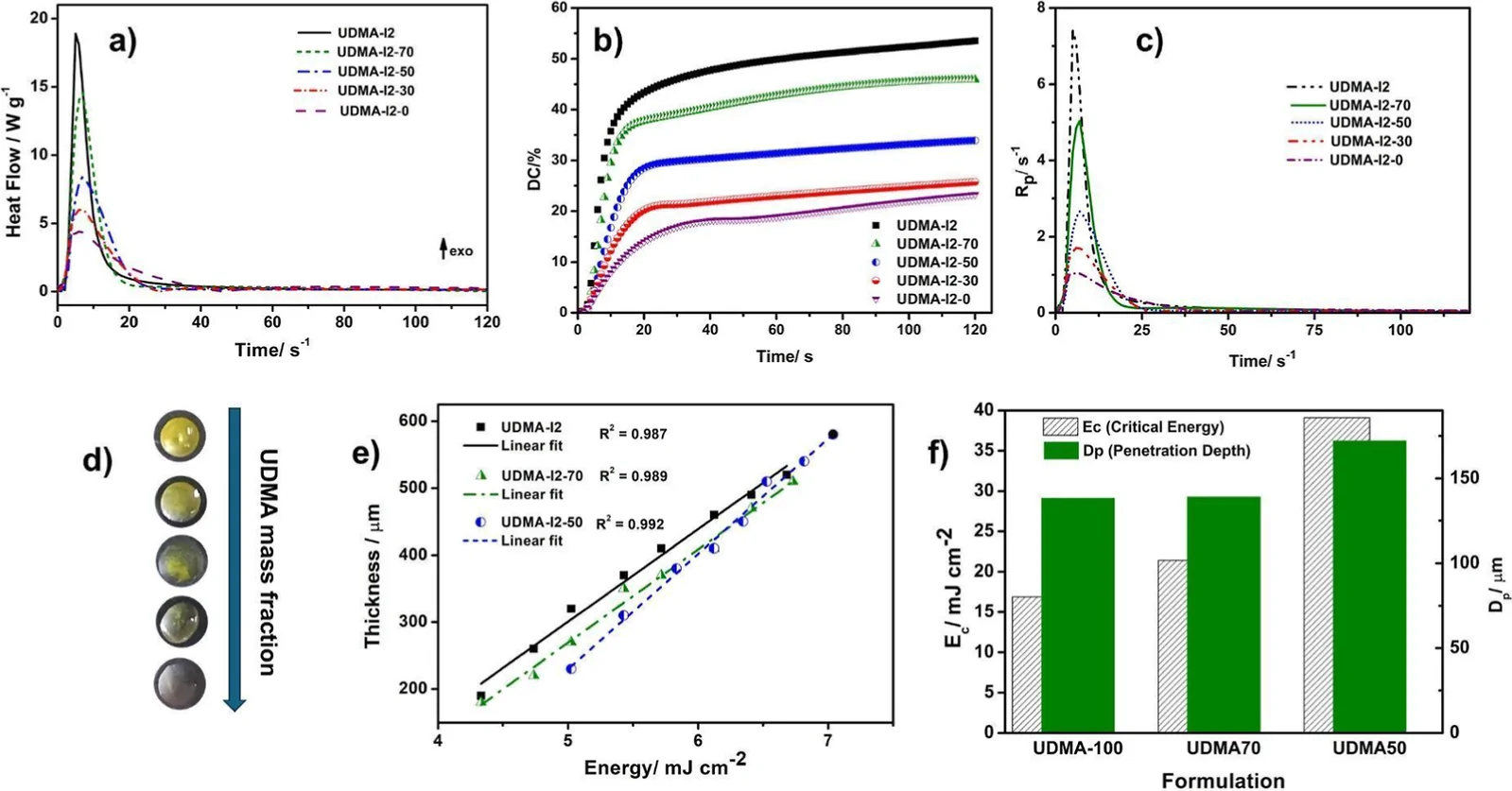
Results related to the formulations varying the UDMA/TEGDMA mass proportion, keeping the concentration of **Q-SMe** and EDB: (a) DSC curves, (b) degree of conversion of monomers into polymer (DC/%), (c) rate of polymerization (*R*_p_) obtained by photo-DSC experiments, and (d) polymers obtained at the end of experiments. (e) Working curves and (f) their respective critical energy (*E*_c_) and penetration depth (*D*_p_).

**Table 2:** Results Obtained by Photo-DSC for the Different Formulations between UDMA and TEGDMA

formulation	photo-DSC results^a^				TG/DTG results		
	Δ*H*_T_ (J g^–1^)	*R*_p_ (s^–1^)	PT (s)	DC (%) after 120 s	*T*_therm. estb_/°C	greatest MDR/% min^–1^	greatest *T*_MDR_/°C
UDMA-I2	198.3	7.4	5	53.5	240.0	10.1	431.0
UDMA-I2–70	121.4	5.0	7	47.9	176.0	8.2	428.0
UDMA-I2–50	104.8	2.6	7	33.9	172.0	9.0	310.0
UDMA-I2–30	65.5	1.7	6	25.8	165.0	9.7	294.0
UDMA-I2–0	94.7	1.0	6	23.3	153.0	5.6	254.0

a Data calculated by photo-DSC curves, eqs 2 and 3. ΔH_T_: total enthalpy variation; PT: peak temperature; DC (%): degree of polymerization of monomers into polymers. *T*_therm. estb_: temperature of thermal stability; Greatest MDR: maximum degradation rate of analysis, and greatest *T*_MDR_: temperature of maximum degradation rate of analysis.

These five systems were analyzed by TG/DTG-DTA, and the resulting curves are presented in Figure S25. As expected, systems containing only UDMA (UDMA-I2) or only TEGDMA (UDMA-I2–0) presented thermal behavior without significant differences compared to those reported in the literature for systems based on camphorquinone or other quinoline derivatives.^9,12,41^ However, as the TEGDMA content increased in the formulations, a decrease in thermal stability was observed (Table 2). This behavior suggests that although dimethacrylate monomers can undergo thermal polymerization during heating, the incorporation of TEGDMA into UDMA-based systems, in the presence of the photoinitiating system **Q-SMe**/EDB, leads to a reduction in cross-linking density during photopolymerization. This is supported by the temperature maximum degradation rate (*T*_MDR_), which shifts to lower values as TEGDMA content increases. The detailed data related to each sample’s thermal decomposition are presented in Table S7.

Therefore, based on these results, only the formulations UDMA-I2–0, UDMA-I2–70, and UDMA-I2–50 were kept for the Jacobs working curves. The resulting working curves are presented in Figure 7e, while the values of critical energy (*E*_c_) and penetration depth (*D*_p_), obtained using eq 4, are shown in Figure 7f. Increasing the TEGDMA content increases, the viscosity of the medium decreases, and *D*_p_ values are enhanced from 138.3 to 172.2 μm, indicating greater light penetration through the resin. However, the energy (*E*_c_) also increased from 16.9 to 39.1 mJ cm^–2^, suggesting that a higher exposure is required to initiate the formation of a cured layer. Therefore, increasing the TEGDMA content enhances light penetration while demanding higher critical energy for curing. Based on its photo-DSC performance and intermediate *E*_c_ and *D*_p_ values, UDMA-I2–70 was selected for the vat photopolymerization 3D-printing tests, as it combined moderate critical energy with sufficient light penetration.

### Characterization of Resulting Materials from Vat 3D Printing Using a Quinoline Derivative as PIS

Figure 8 presents the 3D printed samples, which appear yellow due to the quinoline derivative **Q-SMe**. The first sample is a Celtic flower with well-defined surface details (Figure 8a), which are highlighted under UV light (Figure 8b). Figure 8c,d present the micrographs and show the good resolution of the printed sample, as well as the layered structure. Figure 8e presents the honeycomb, demonstrating the good performance of PIS (**Q-SMe**/EDB) in printing a more complex object, as shown in Figure 8f,g. The honeycomb structure was then sliced to analyze the middle section in order to better visualize the layers. The resulting micrograph is presented in Figure 8h, where the layers display homogeneous space and a thickness close to the target height (0.05 mm). In addition, the printing objects show no evidence of overcuring, demonstrating the strong potential of the quinoline derivative **Q-SMe** as a photoinitiator for 3D vat photopolymerization.

**Figure 8:**
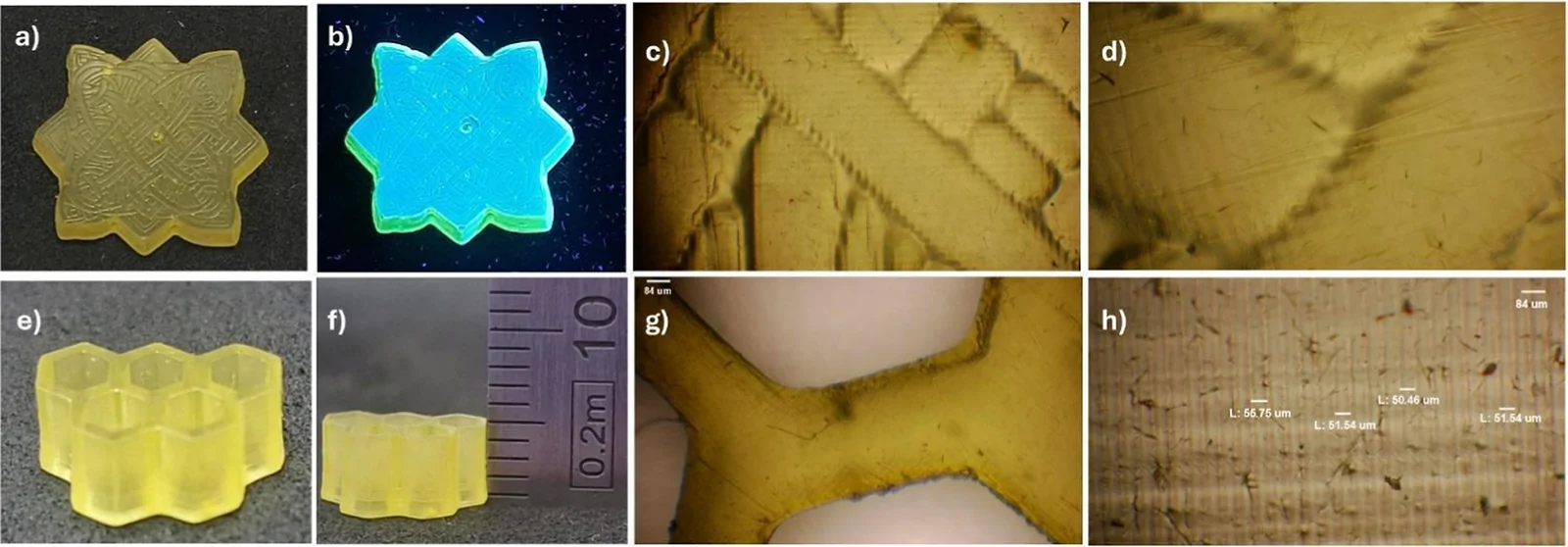
Objects obtained using the formulation UDMA-I2–70 in vat photopolymerization Celtic flower (first line) under (a) ambient light, (b) UV light. (c,d) Micrographs at different magnifications. Honeycomb (second line): (e) superior and (f) lateral views. (g,h) Micrographs at different magnifications.

Using the identical printing parameters, the same objects were printed applying the formulation UDMA-K2–70. The resulting images and TG/DTG-DTA curves are present in Figures S26 and S27, respectively. Although the final material presented thermal stability equal to 228.0 °C and thermal behavior similar to the previous materials, the final materials are overcured, as their edges lack definition and no holes appear in the honeycomb structure. Therefore, applying the CQ/EDB system under the same conditions results in materials with less resolution compared to those printed using SMe/EDB.

## Conclusion

This study demonstrates that quinoline derivatives are effective visible-light photoinitiators for UDMA-based photopolymerization, with their performance strongly influenced by both molecular structure and formulation composition. Blue-light irradiation was significantly more effective than UV irradiation, resulting in higher monomer conversions. Among the evaluated photoinitiating systems, the combination of Q-SMe and EDB exhibited the best overall photopolymerization performance. Statistical analysis confirmed that both the photoinitiating system and photoinitiator concentration significantly affected the final monomer conversion. Photolysis, UV–vis spectroscopy, and mass spectrometry provided evidence for the formation of a sulfoxide intermediate during Q-SMe photoactivation, supporting the proposal that this derivative can operate through both type I and type II photoinitiation pathways, whereas the photochemical behavior of Q-OMe is consistent with a predominantly type II mechanism. The incorporation of TEGDMA as a reactive diluent increased the penetration depth while reducing the thermal stability and cross-linking density of the resulting polymers. Finally, the optimized Q-SMe/EDB formulation enabled the fabrication of well-defined 3D printed structures with improved dimensional fidelity compared with the conventional CQ/EDB system under identical printing conditions, highlighting its potential as an alternative visible-light photoinitiating system for vat photopolymerization.

## Supplementary Material



## Notes

Experimental procedures; synthesis and characterization of quinoline photoinitiators (^1^H NMR, FTIR, and mass spectrometry); TG-DTA and DSC analyses; UV–vis absorption spectra and molar extinction coefficients; complementary MIR photopolymerization data and statistical analyses; UV–vis photolysis and photoproduct mass spectrometry; comparison with the CQ/EDB photoinitiating system; thermal characterization of the obtained polymers; and additional vat photopolymerization results (PDF)
