# Supplementary material for: Quinoline-based Photoinitiators for Vat Photopolymerization: Synthesis and Formulation Design for 3D Printing
Source: ACS Polym Au. 2026 Aug 12;6(5):1376–88. doi: 10.1021/acspolymersau.6c00101 (PMC13673911; doi:10.1021/acspolymersau.6c00101)
Supplement: Supplementary file 1 [file lg6c00101_si_001.pdf]

Supplementary Information

Quinoline-Based Photoinitiators for Vat Photopolymerization: Synthesis and Formulation Design for 3D Printing

Ilana S. Rosário[a], Caroline Gaglieri[a], Vinicius H. de Carvalho[a], Emily B. de Barros[a], João A. R. Bassetto[a], Gabriela Cremasco[a], Gilbert Bannach[a], Luiz C. da Silva-Filho[a]*.

[a]*Universidade Estadual Paulista (UNESP), Faculdade de Ciências, Bauru, 17033-260, Brazil*

Experimental

All chemicals and solvents were purchased from Sigma–Aldrich (St. Louis, MO, USA) and used without further purification Thin-layer chromatography was performed on 0.2 mm Merck 60F254 silica gel aluminum sheets, which were visualized with a vanillin/methanol/water/sulfuric acid mixture. Infrared (IR) spectra were recorded on a Bruker Vertex 70 Fourier Transform Spectrometer using a Bruker Platinum ATR unit. Bruker DRX 400 spectrometer was employed for the NMR spectra ($CDCl_3$ solutions) using tetramethylsilane as internal reference. MS analyses were recorded with an LCMS-IT Mass Spectrometer (THERMO). The melting points of synthesized compounds were determined by Differential Scanning Calorimetry analyses (DSC).

General procedure for the synthesis of quinoline derivatives

1.0 mL of a 0.5 M hydrochloric acid solution was added to a round-bottom flask containing a benzaldehyde derivative (1.0 mmol), an aniline derivative (1.0 mmol) and phenylacetylene (1.0 mmol). Initially a suspension occurred, but as the reaction pot was heated, the reagents solubilized. The reaction occurred under reflux and stirring for 72 hours. The completion of the reaction was verified by monitoring the consumption of the formed intermediates by thin layer chromatography (hexane:ethyl acetate 3:2). The reaction mixture was quenched with water (5.0 mL) and ethyl acetate (5.0 mL) and the pH of the solution was neutralized using a 2.0 M sodium hydroxide solution. The organic phase was extracted with ethyl acetate (3 x 10 mL) and washed with a saturated sodium chloride solution (10 mL) and dried over anhydrous magnesium sulfate. The solvent was recovered after vacuum evaporation and the organic mixture was dissolved in boiling methanol (5 to 10 mL) that, upon cooling, resulted in a yellowish solid.

2-(4-methoxyphenyl)-6-nitro-4-phenylquinoline (**Q-OMe**)

Pale yellow solid. ^{1}H-NMR (400 MHz, $CDCl_3$): δ = 8.83 (d, *J* = 2.5 Hz, 1H), 8.47 (dd, *J* = 9.3, 2.5 Hz, 1H), 8.28 (d, *J* = 9.3 Hz, 1 H), 8.24 (dd, *J* = 7.0, 2.3 Hz, 2 H), 7.64–7.56 (m, 5 H), 7.07 (dd, *J*= 7.0, 2.3 Hz, 2 H), 3.91 (s, 3 H) ppm; IR (neat): ν max = 748, 958, 1031, 1249, 1467, 2829, 2935 cm-1; MS (m/z) = 357,20

2-(4-(methylthio)phenyl)-6-nitro-4-phenylquinoline (**Q-SMe**)

Yellow solid. ^{1}H-NMR (400 MHz, $CDCl_3$): δ 8.84 (d, *J* = 2.5 Hz, 1H), 8.49 (dd, *J* = 9.3, 2.5 Hz, 1H), 8.33 (d, *J* = 9.3 Hz, 1H), 8.20 (m, 2H), 7.95 (s, 1H), 7.63-7.55 (m, 5H), 7.41 (m, 2H), 2.57 (s, 3H) ppm; IR (neat): *ν* max= 754, 810, 1026, 1186, 1479, 1589, 2919 cm-1. MS (m/z) = 373.15

Figure S1. ^{1}H-NMR Spectrum (400 MHz, $CDCl_3$) of compound **Q-OMe**.

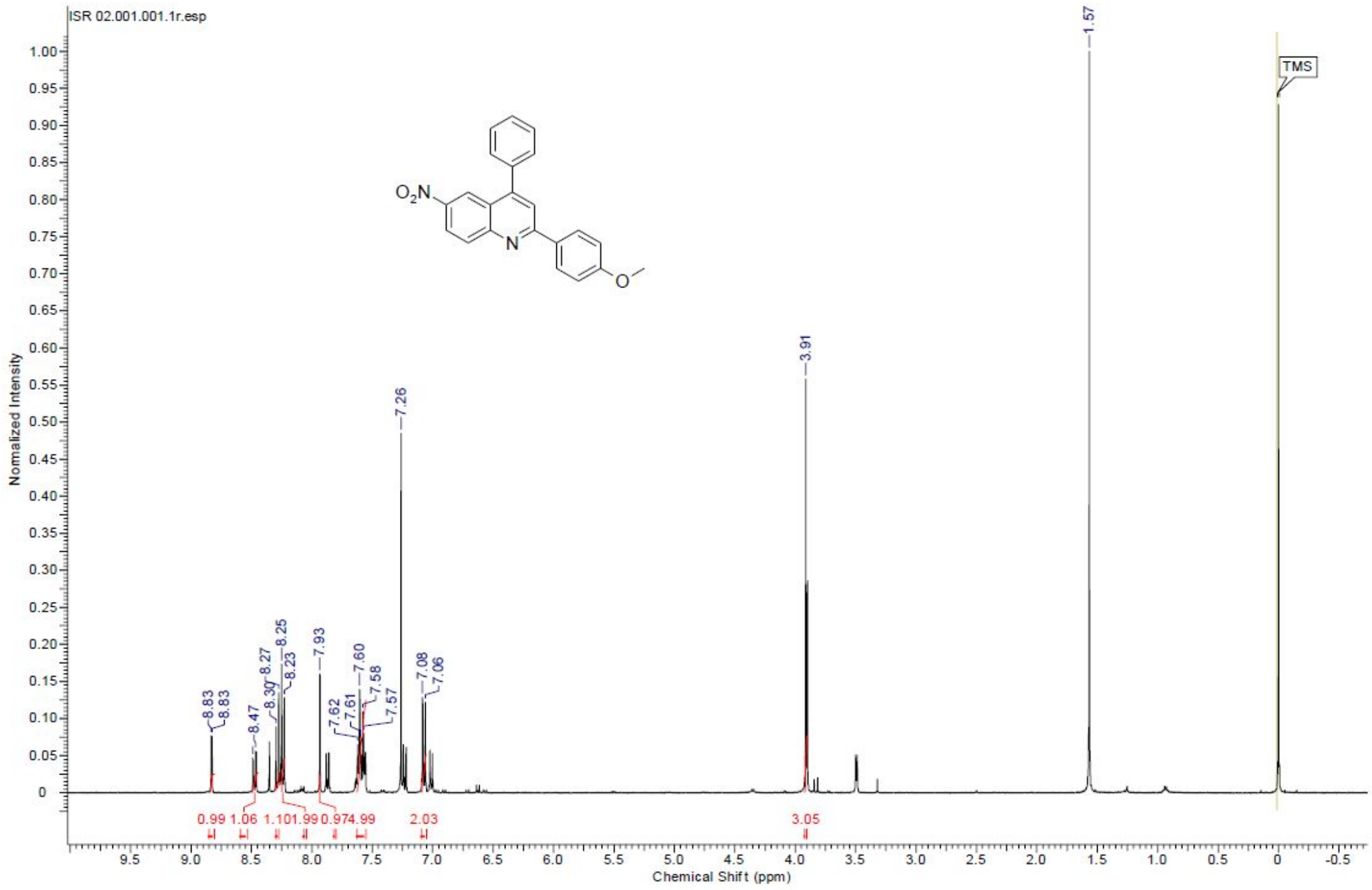

Figure S2. ^{1}H-NMR Spectrum (400 MHz, $CDCl_3$) of compound **Q-SMe**.

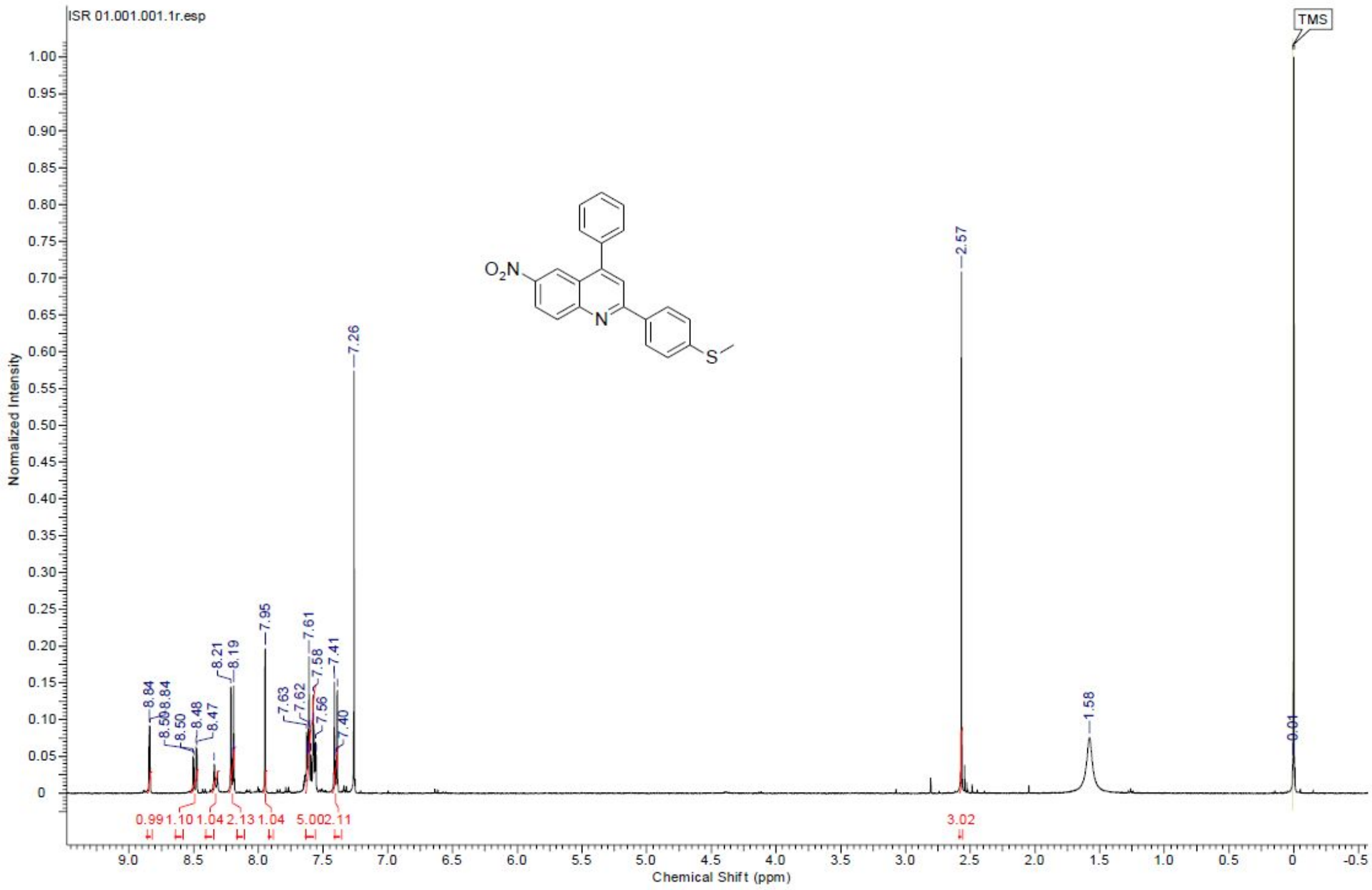

Figure S3. Mass spectrum of compound **Q-OMe**

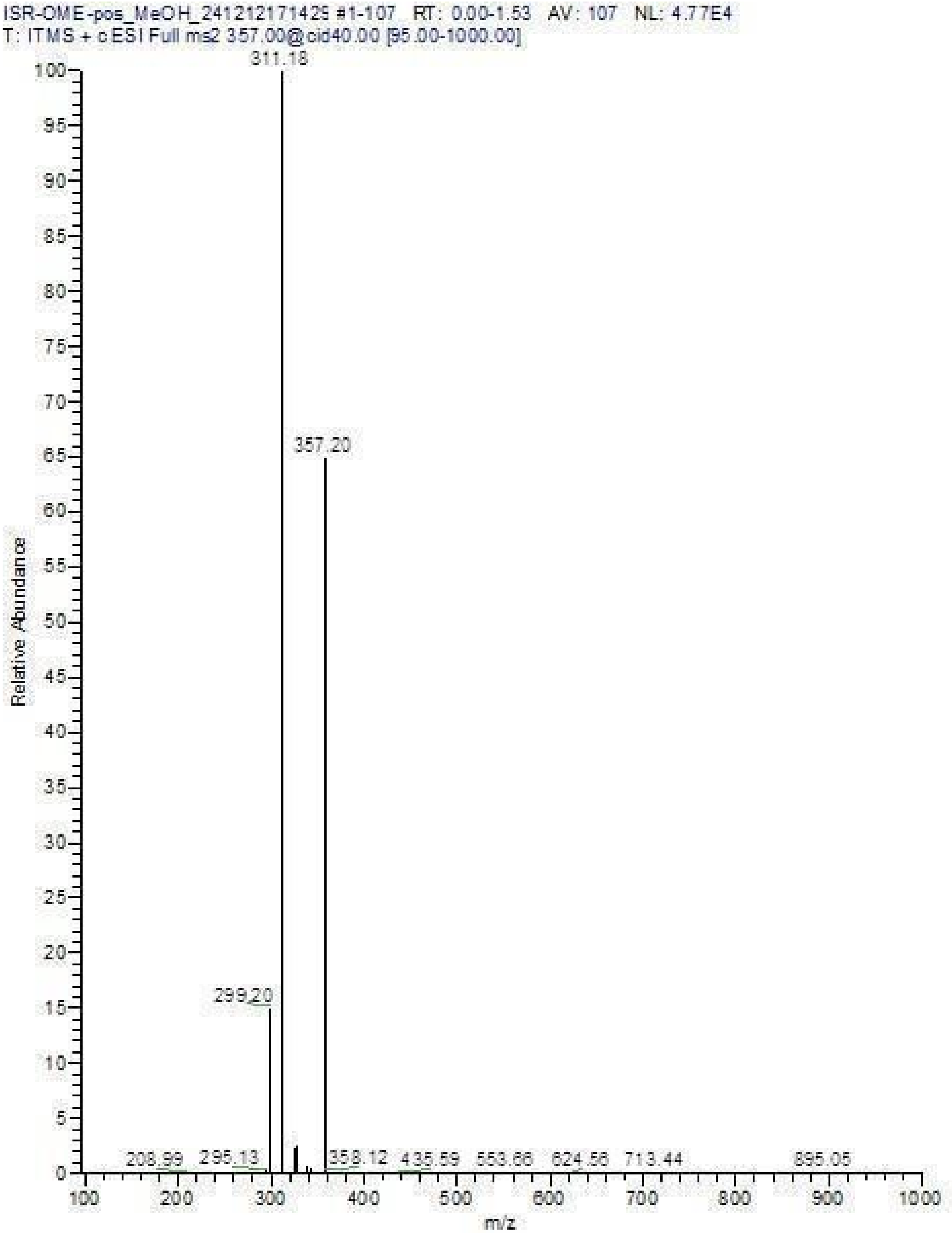

Figure S4. Mass spectrum for compound **Q-SMe**

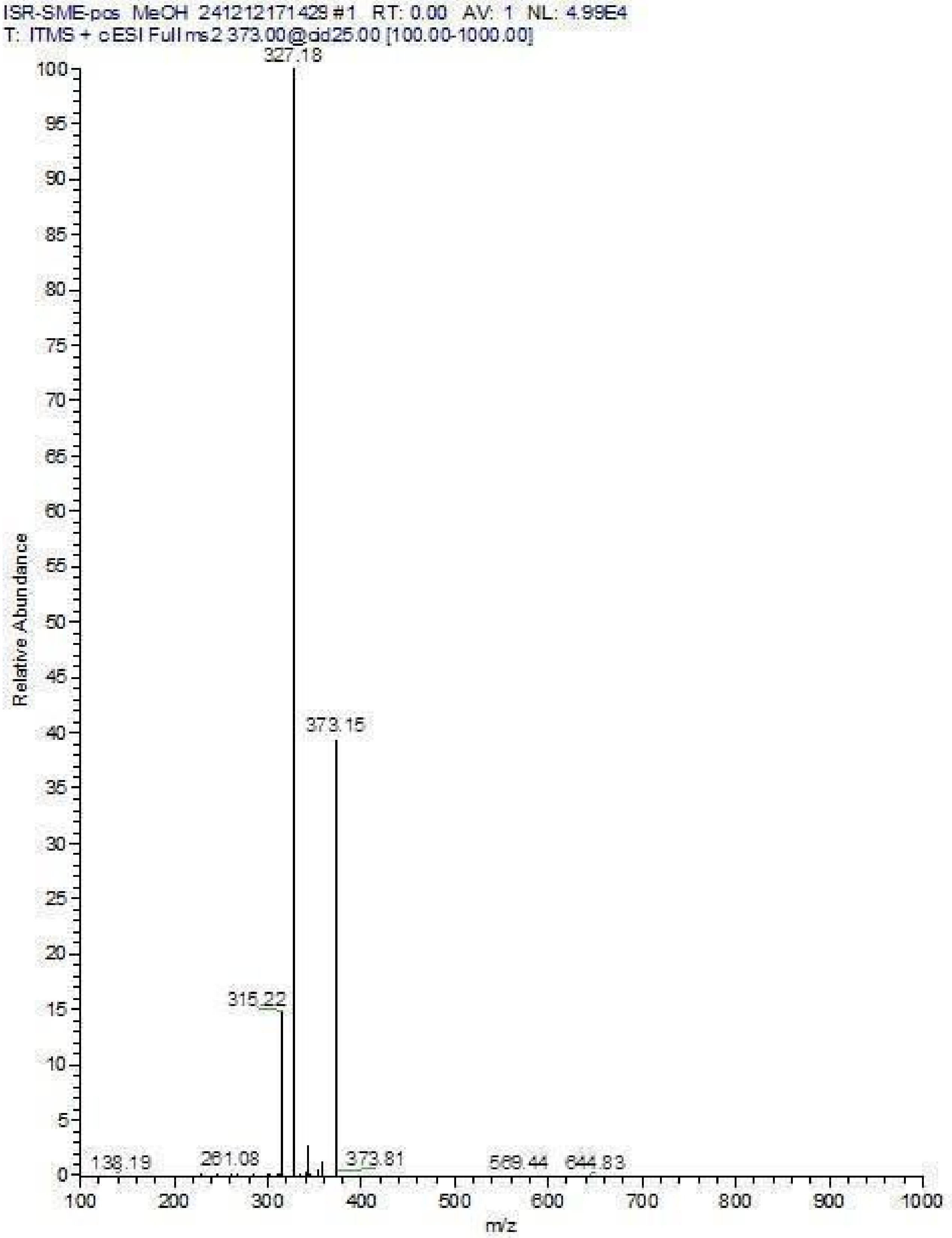

Figure S5. FTIR (ATR) spectrum of compound Q-OMe.

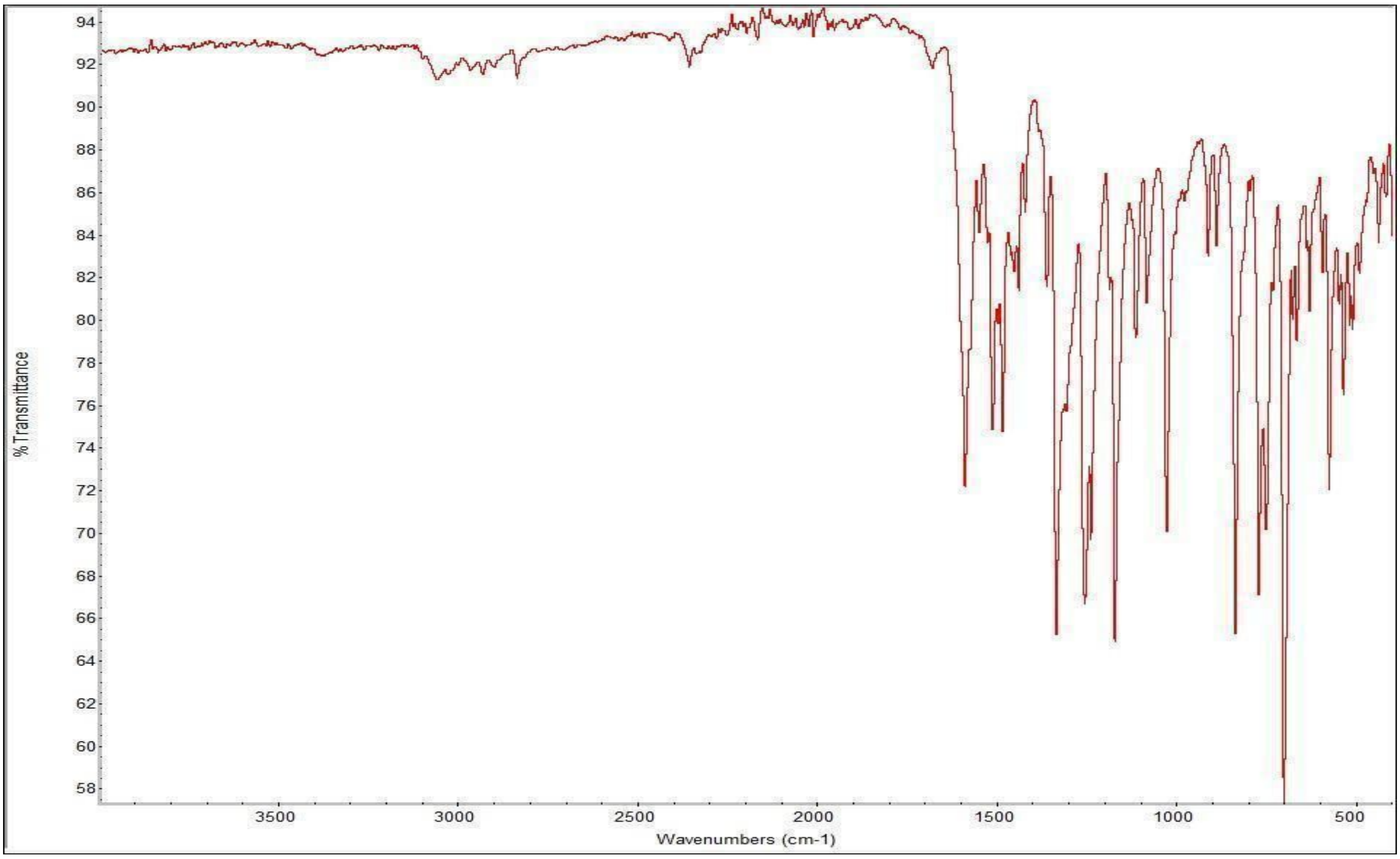

Figure S6. FTIR (ATR) spectrum of compound Q-SMe.

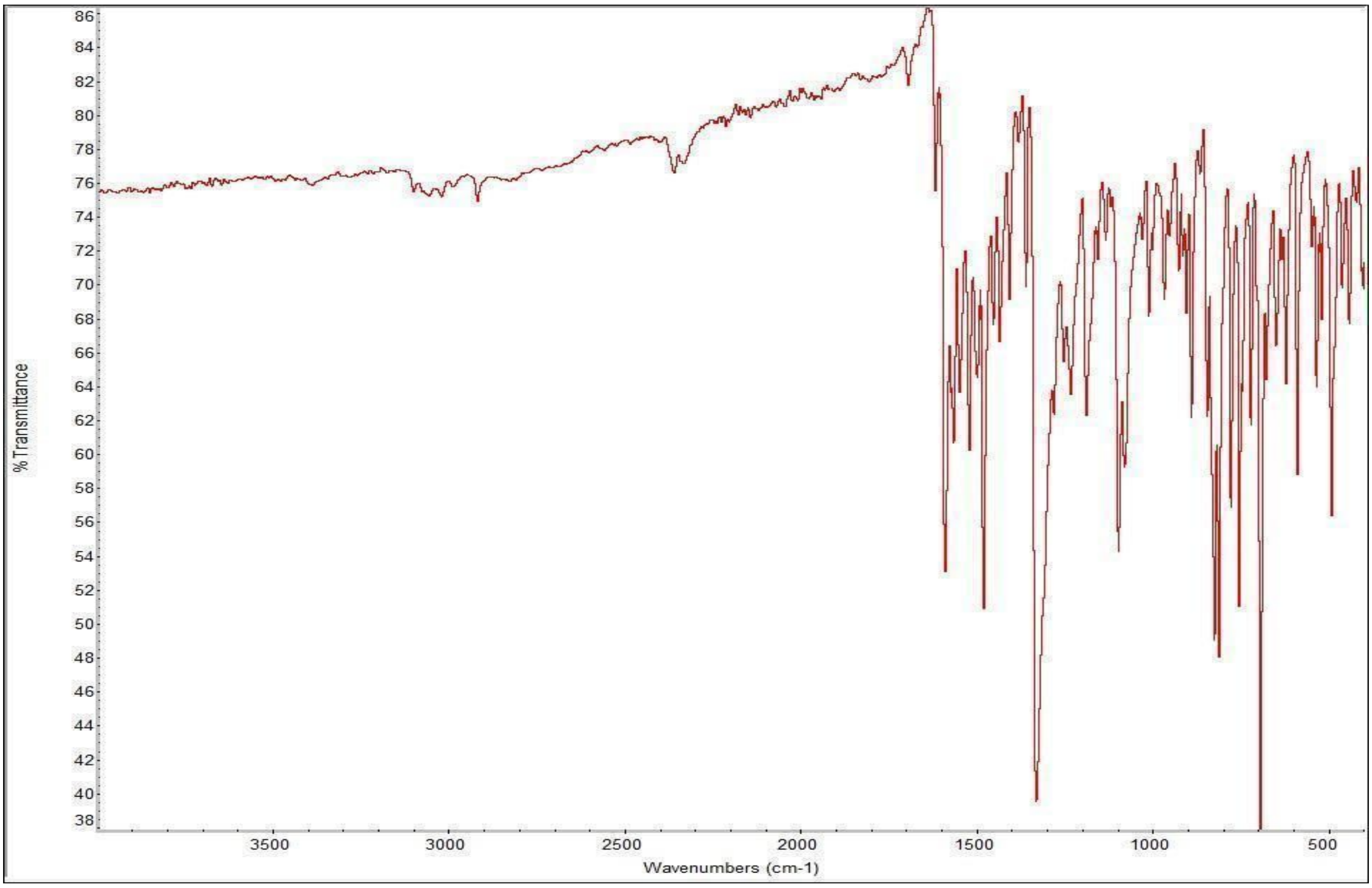

Thermal Analysis of Quinoline Derivatives

Q-OMe (Figure 1c) was thermally stable until 270.0 ºC and decomposed in two consecutive steps of mass loss, as evidenced by its DTG curve. An endothermic peak at 218.0 ºC was observed in the DTA curve, without simultaneous mass loss in the TG curve, which is attributed to the sample melting (confirmed by visual tests). In addition, both steps of mass loss resulted in exothermic peaks in the DTA curve, as observed in Table S2, which exhibits the temperature interval, mass loss value, as well as the events observed in the DTA and DTG curves that occurred simultaneously with each step of mass loss.

The sample Q-SMe (Figure 1c) exhibited a thermal behavior different from Q-OMe. It presented four consecutive steps of mass losses (Table S2), and lower thermal stability (180.0 ºC). This first mass loss (Δm= 1.73%) occurred simultaneously with an endothermic peak in the DTA curve (T_p = 197.0 ºC) and it was suggested to be resulted from the evaporation of a small portion of residual reagent, which evaporated when the quinoline melted (the melting process was corroborated by visual tests). The second mass loss in TG curve resulted in an exotherm event in the DTA curve and can be considered a complex step due to the consecutive and overlapped peaks in the DTG curve. After this, the sample lost mass continuously and did not result in visible exo or endothermic events in the DTA curve, nor peaks in the DTG curve. Finally, the last mass loss occurred at higher temperature intervals compared to Q-OMe (Table S2), suggesting that although Q-SMe started to lose mass before, the degradation process occurred slowly and in a different way than the observed to Q-OMe, which is corroborated by the lower values of maximum degradation rate (MDR) of Q-SMe throughout the mass losses (Table S2).

The DSC curves of quinolines are presented in Figure 1d. The sample Q-OMe exhibited an exothermic event (T_p = 159.0 ºC and ΔH = 10.3 J g^{-1}), which could be associated with a possible crystalline change. Afterward, an intense and sharp endothermic event was observed due to the absence of mass loss in its TG curve (Figure 1c), and by visual tests, it was attributed to the sample melting ($T_{p\ melt}$ = 217.4 ºC and $\Delta_{melt}H$ = 133.0 J g^{-1}). Based on its melting process, it was possible to verify the elevated purity of quinoline obtained in this work: 98.8%. To avoid mass release inside

DSC equipment, the sample Q-SMe was previously heated in the TG equipment (until 220 ºC) and then cooled at room temperature. Then, this sample was submitted to DSC analysis, and the resulting curve is presented in Figure 1d. It exhibited an exothermic event during its heating (93.0-135.0 ºC and ΔH = 5.7 J g^{-1}), it was also suggested that a crystalline change in the sample occurred. This is supported by the occurrence of a large endothermic event with a shoulder (pointed by a red arrow in Figure 1d), suggesting that two distinct crystalline forms are melting and resulted in a global enthalpy equal to $\Delta_{melt}H$ = 92.0 J g^{-1}. To confirm that this event did not result from a degradation process, the sample was submitted to other TG-DTA analysis and also collected different MIR spectra (before heating, and after heating). The resulting curves and spectra are shown in Figure S8, respectively. The new thermal stability (220 ºC) was very similar to the beginning of the first mass loss after the release of impurity observed in Figure 5b. In addition, no significant changes were observed in the thermal decomposition profile nor MIR spectra. Thus, it is possible to suggest that there is no degradation process before 220 ºC, and the events in the DSC curve (Figure 1d) are due to crystalline changes, which will demand detailed study in future work.

Figure S7. TG/DTG-DTA curves of quinoline **Q-SMe** after heating it until 220 ºC.

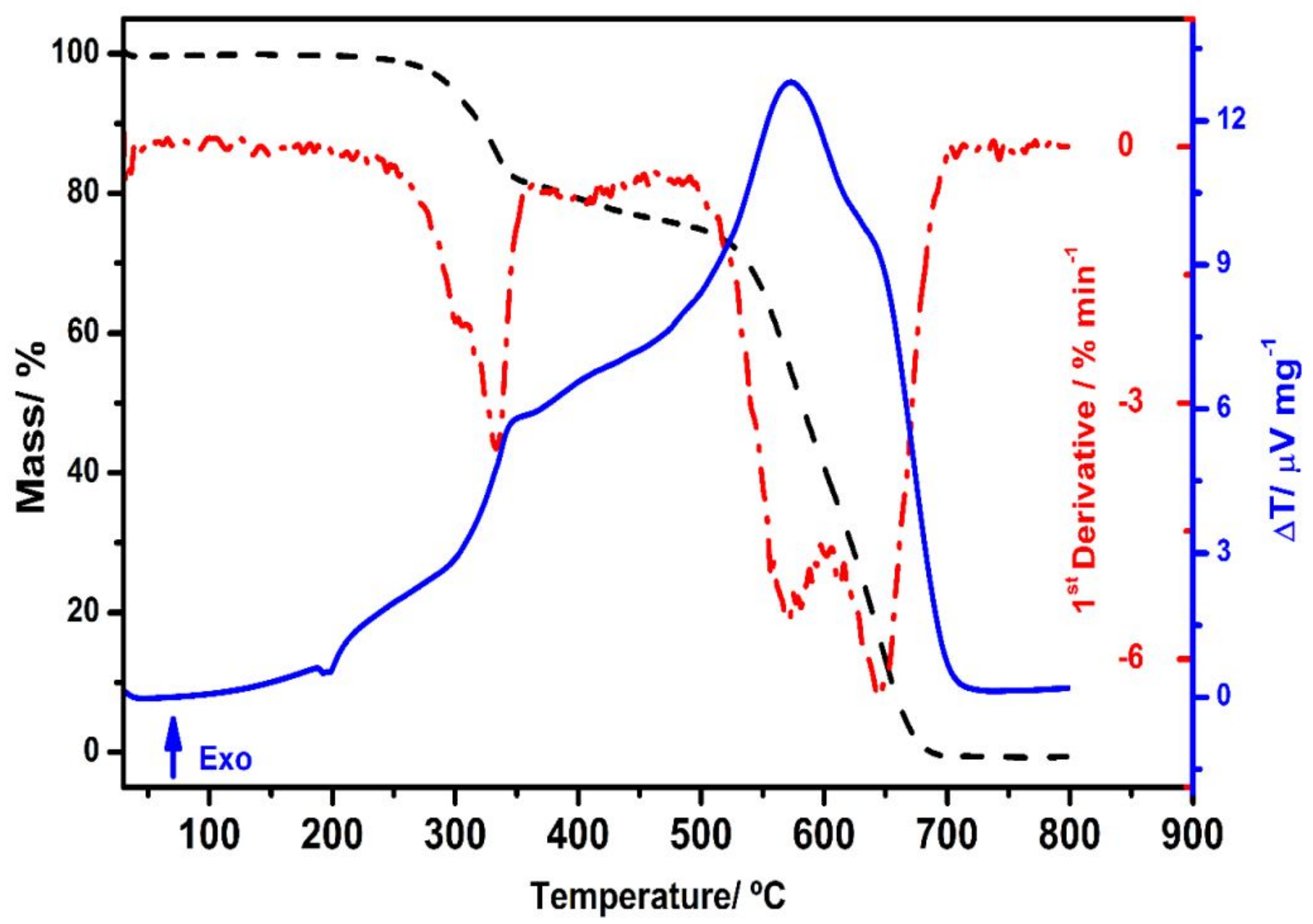

Figure S8. MIR spectra of quinoline **Q-SMe**: *(a)* after synthesis, *(b)* after heating it until 220 ºC, and *(c)* after DSC analysis

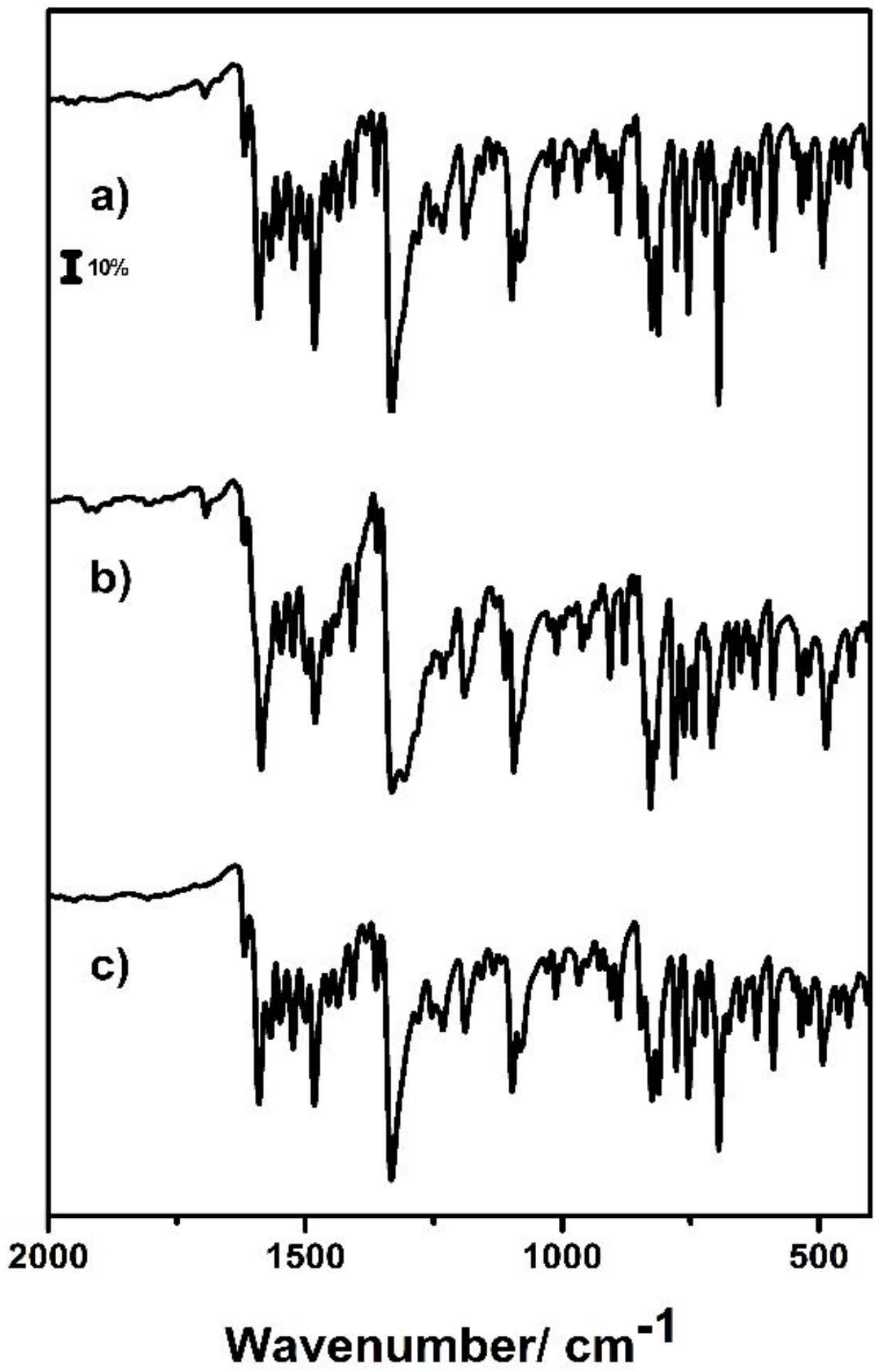

Figure S9. Degree of conversion of monomer into polymer (DC(%)) obtained by MIR for each formulation under UV light.

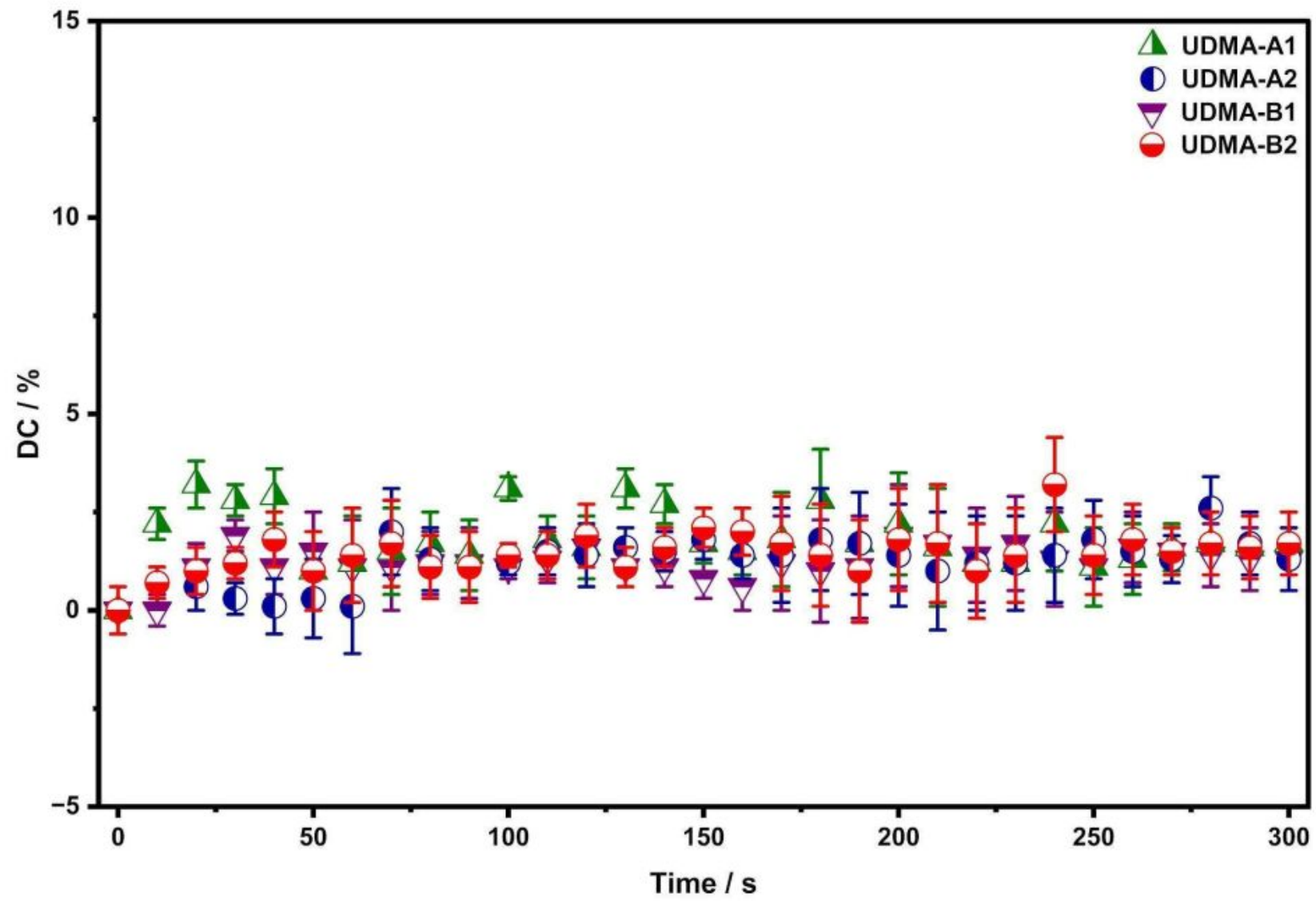

Figure S10. Representative MIR spectra illustrating the decrease of the C=C stretching band during photopolymerization of the formulations: **a)** UDMA-A1 and **b)** UDMA-A2 under blue light.

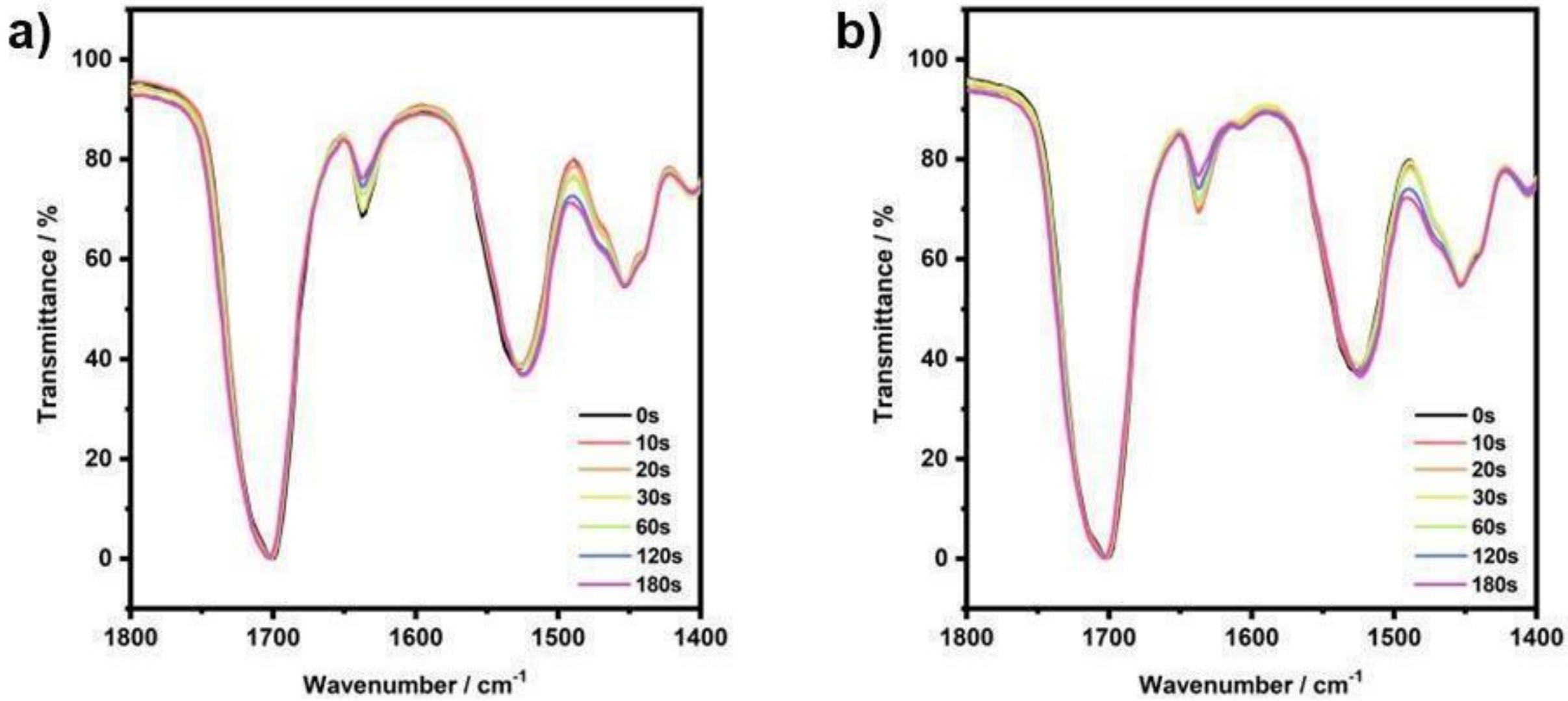

Figure S11. Representative MIR spectra illustrating the decrease of the C=C stretching band during photopolymerization of the formulations: **a)** UDMA-B1 and **b)** UDMA-B2 under blue light.

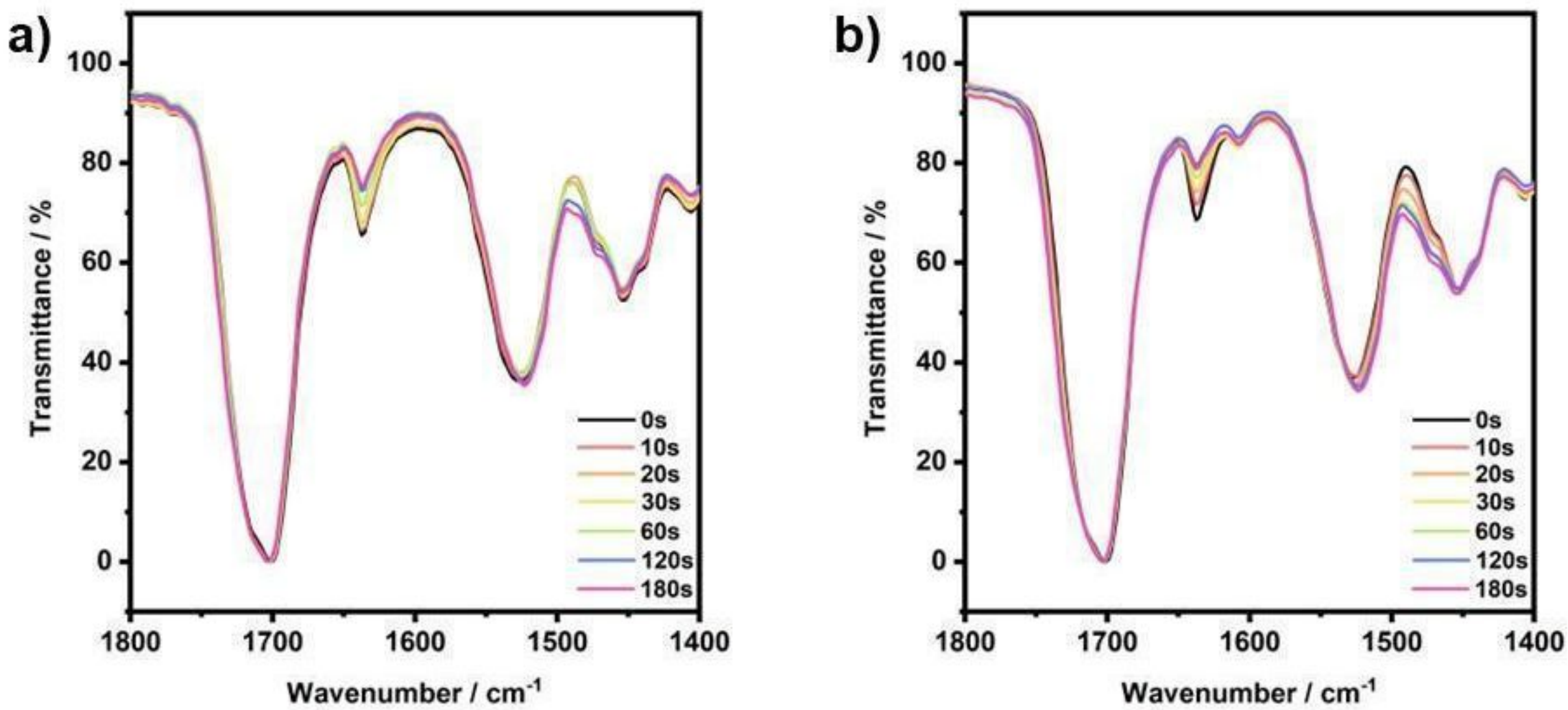

Figure S12. Rate of polymerization (R_p) obtained by MIR for formulations containing *a)* **Q-OMe**, *b)* **Q-OMe**/EDB, *c)* **Q-SMe** and *d)* **Q-SMe**/EDB at different concentrations under blue light.

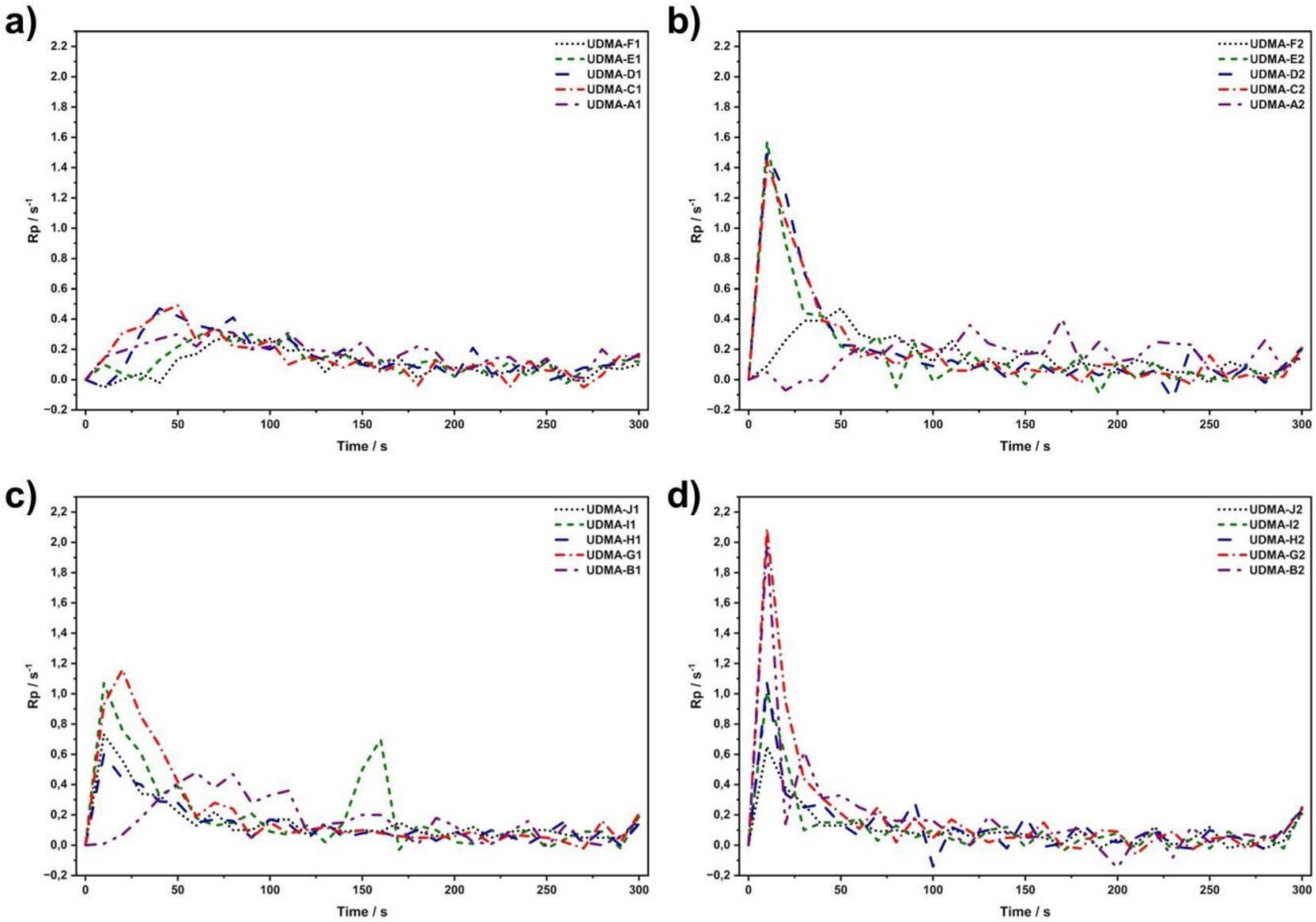

Figure S13. Representative MIR spectra illustrating the decrease of the C=C stretching band during photopolymerization of the formulations: **a)** UDMA-C1 and **b)** UDMA-C2 under blue light.

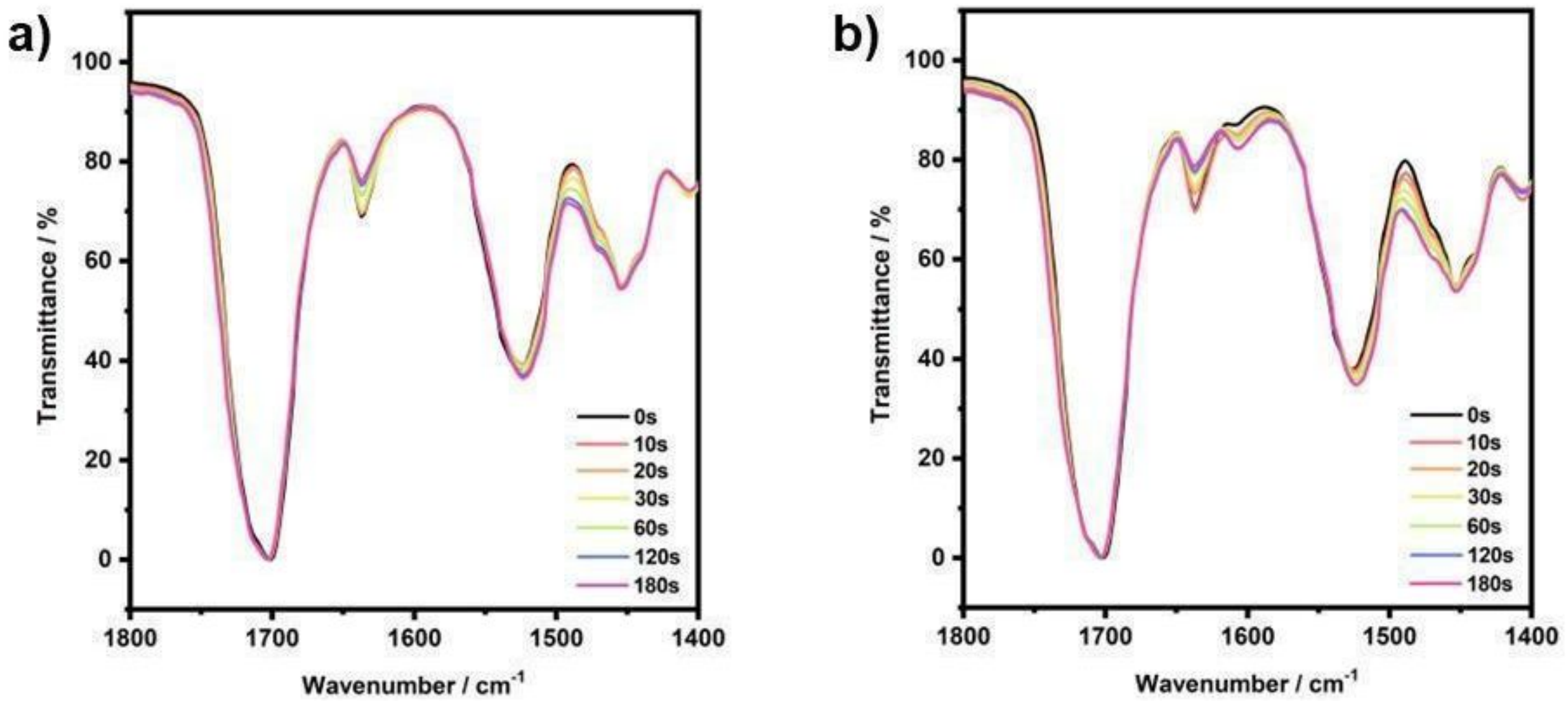

Figure S14. Representative MIR spectra illustrating the decrease of the C=C stretching band during photopolymerization of the formulations: **a)** UDMA-D1 and **b)** UDMA-D2 under blue light.

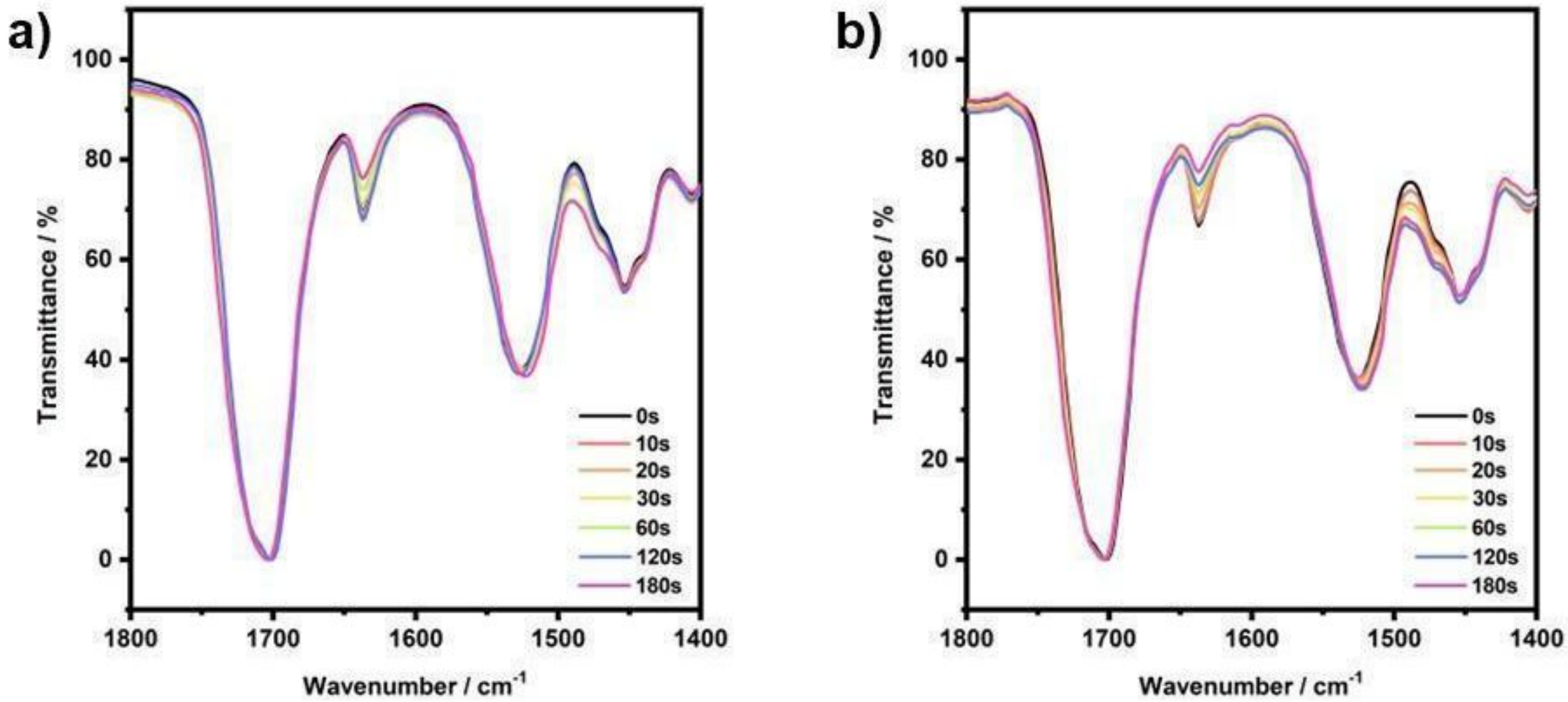

Figure S15. Representative MIR spectra illustrating the decrease of the C=C stretching band during photopolymerization of the formulations: **a)** UDMA-E1 and **b)** UDMA-E2 under blue light.

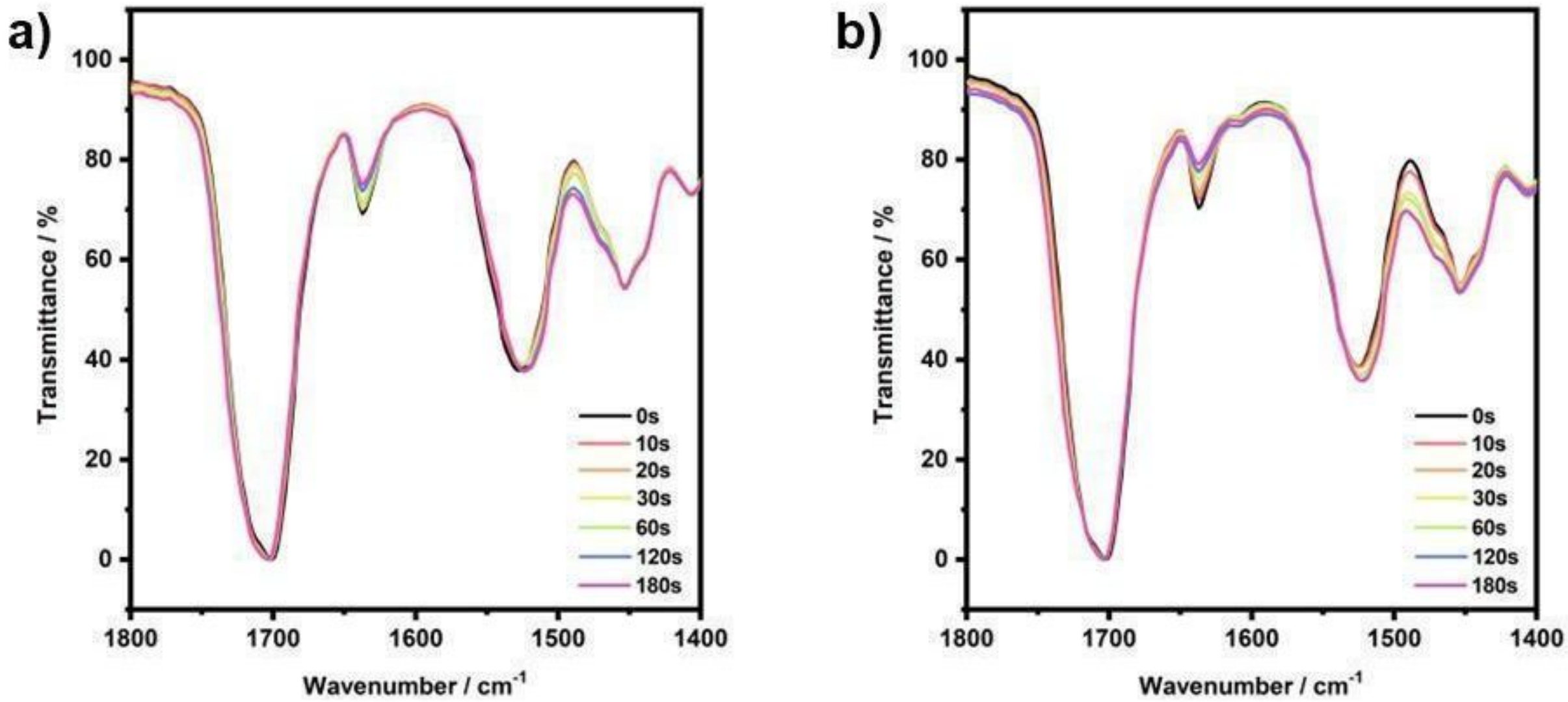

Figure S16. Representative MIR spectra illustrating the decrease of the C=C stretching band during photopolymerization of the formulations: **a)** UDMA-F1 and **b)** UDMA-F2 under blue light.

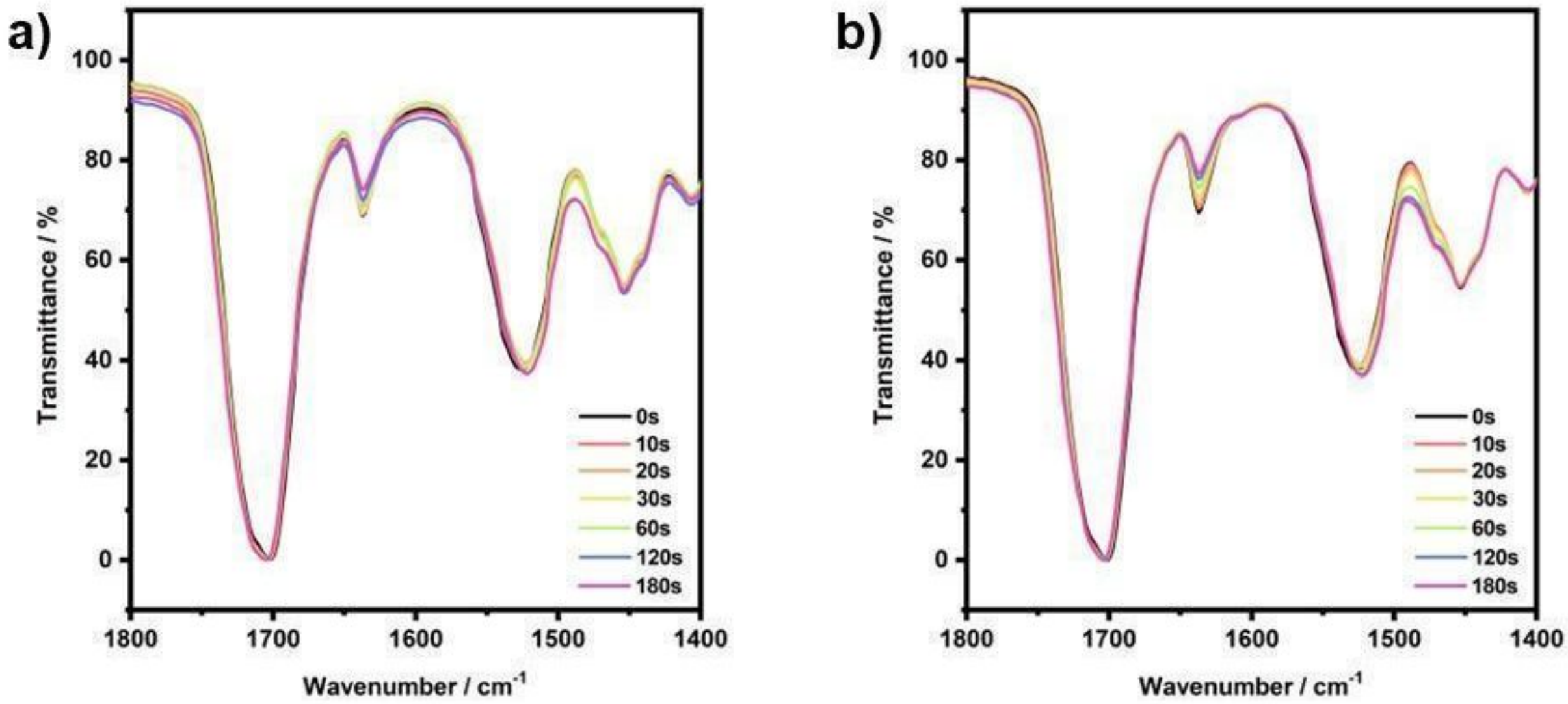

Figure S17. Representative MIR spectra illustrating the decrease of the C=C stretching band during photopolymerization of the formulations: **a)** UDMA-G1 and **b)** UDMA-G2 under blue light.

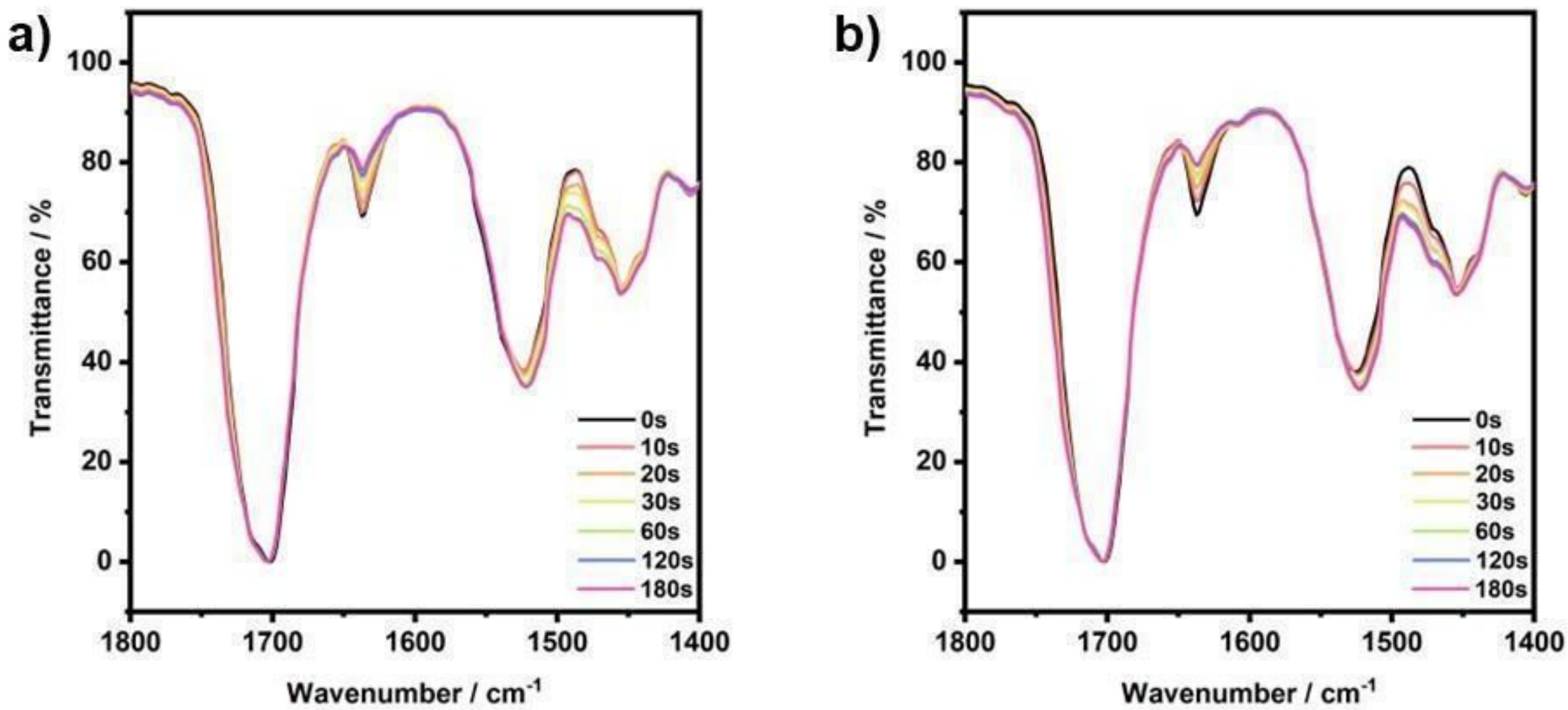

Figure S18. Representative MIR spectra illustrating the decrease of the C=C stretching band during photopolymerization of the formulations: **a)** UDMA-H1 and **b)** UDMA-H2 under blue light.

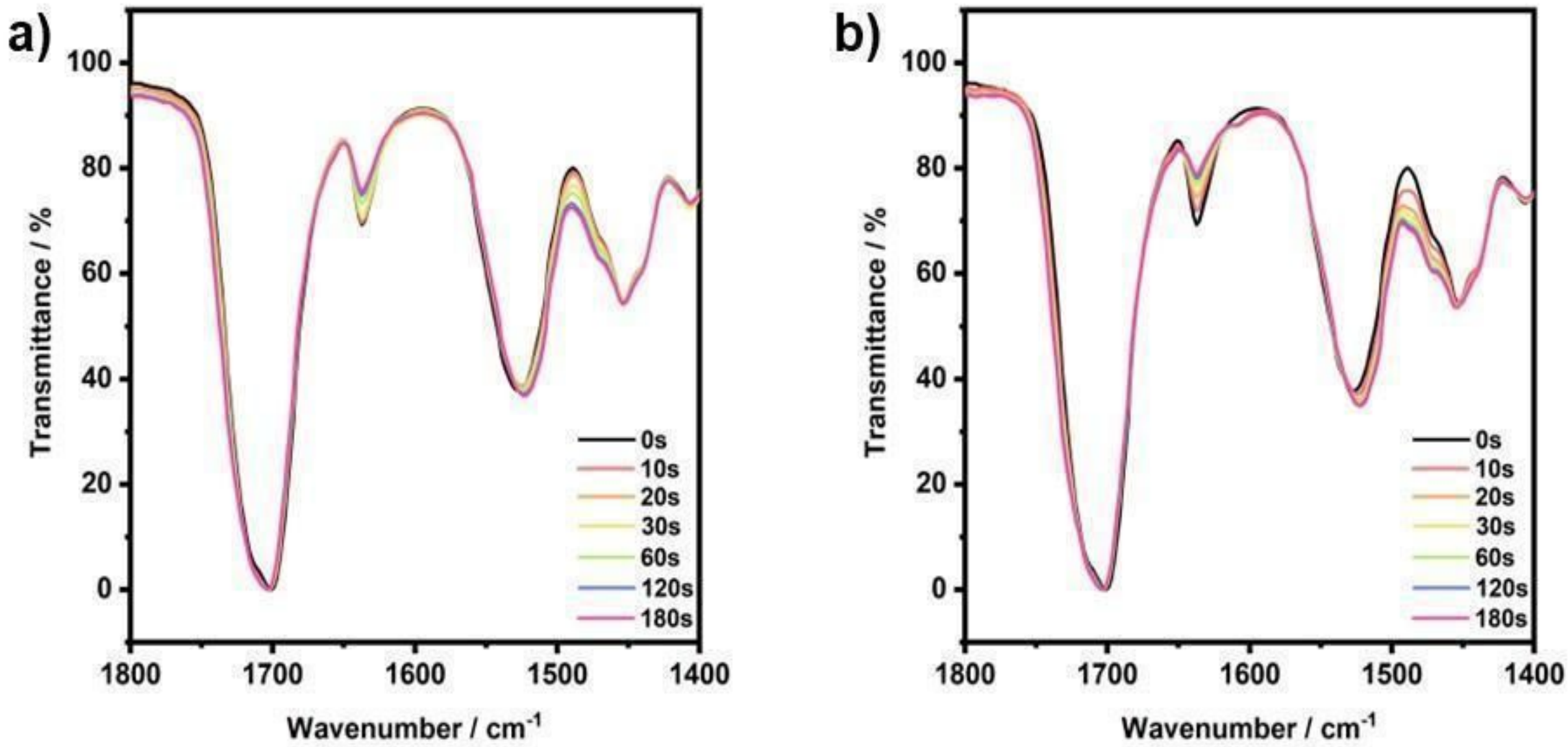

Figure S19 Representative MIR spectra illustrating the decrease of the C=C stretching band during photopolymerization of the formulations: **a)** UDMA-I1 and **b)** UDMA-I2 under blue light.

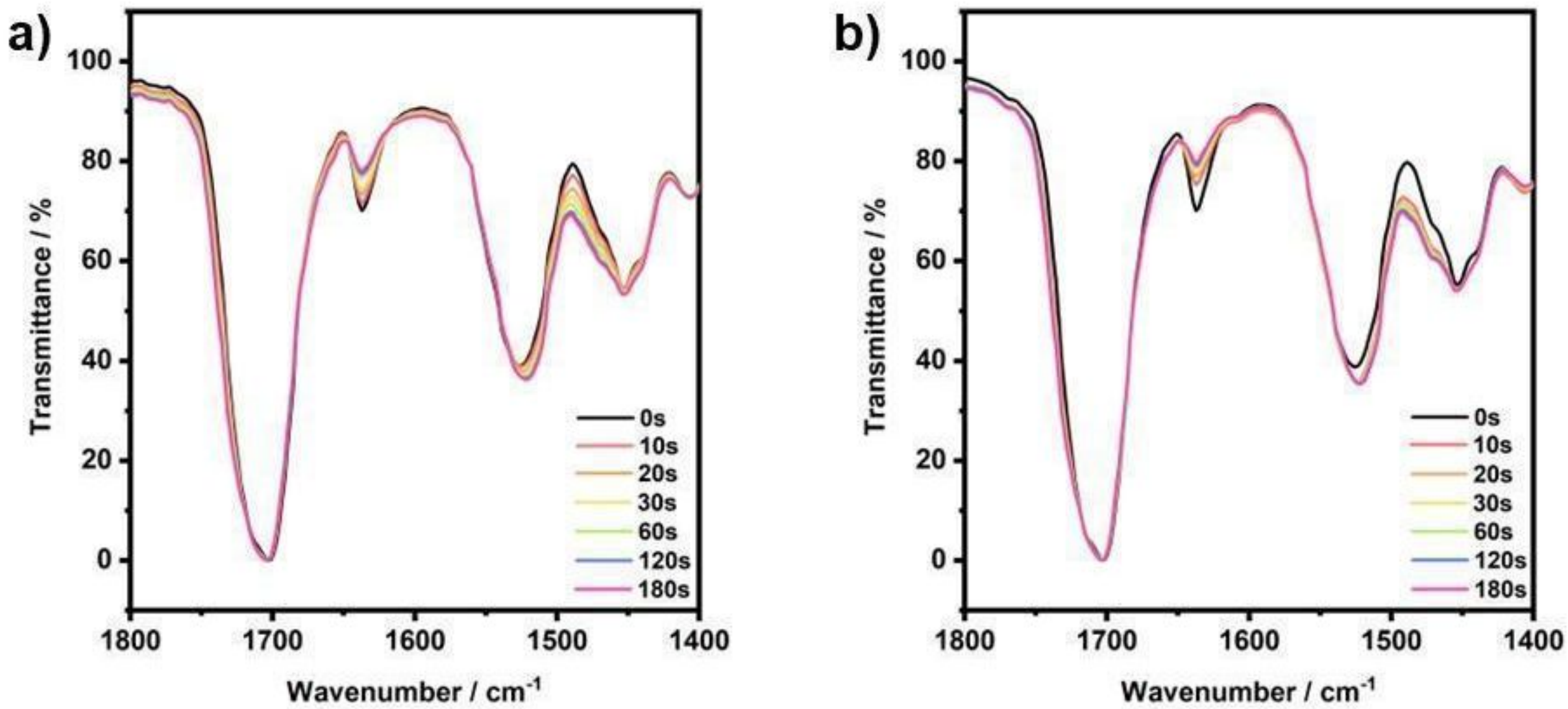

Figure S20. Representative MIR spectra illustrating the decrease of the C=C stretching band during photopolymerization of the formulations: **a)** UDMA-J1 and **b)** UDMA-J2 under blue light.

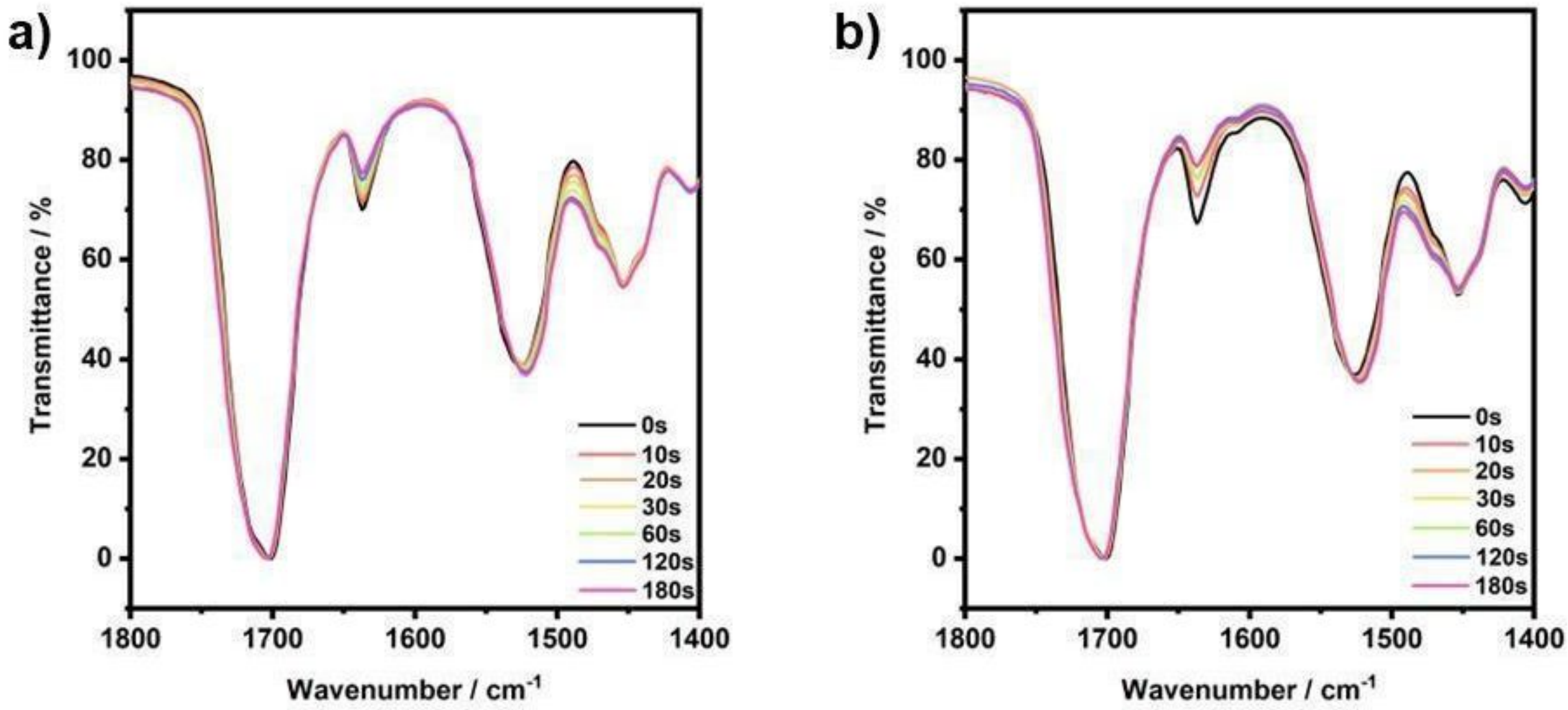

Figure S21. a) Degree of conversion (DC, %) and **b)** polymerization rate (Rp), both determined by MIR, for UDMA/TEG-DMA (70/30 wt%) formulations containing 0.3% of camphorquinone (**CQ**) or quinoline-based (**Q-SMe**) and 0.5% of EDB.

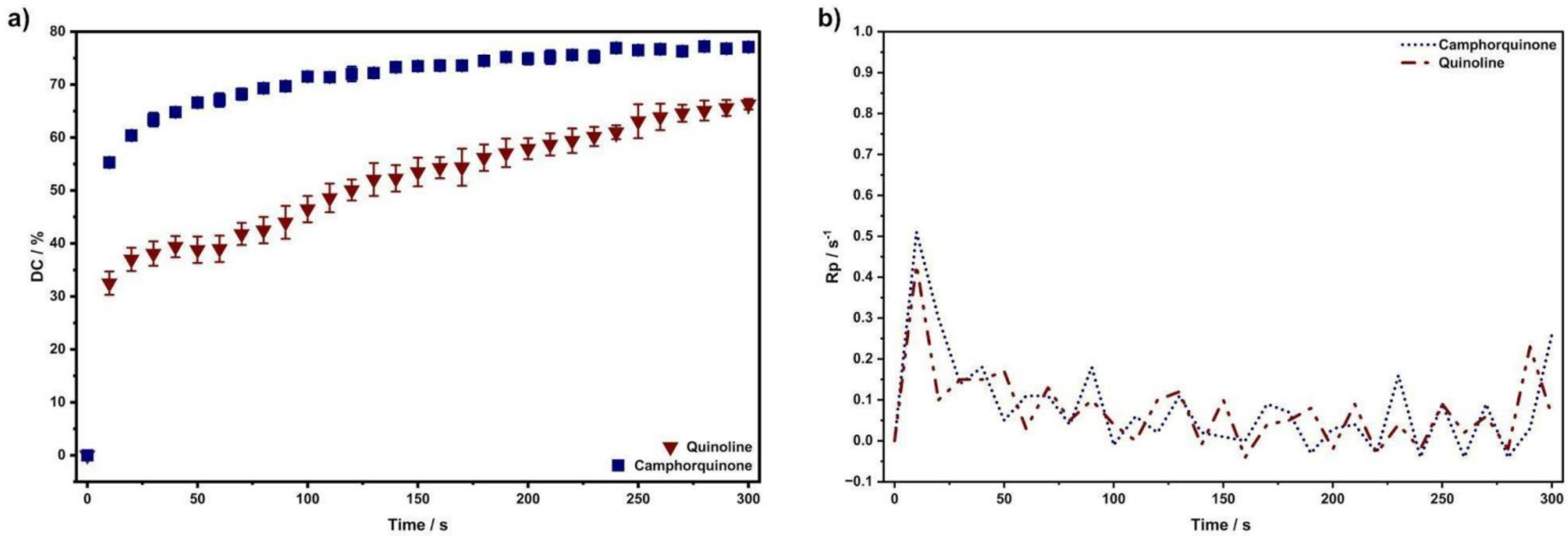

Figure S22. UV-Vis spectra obtained for systems with **Q-OMe** and **Q-SMe**, in the absence and presence of EDB, after different times under Blue irradiation.

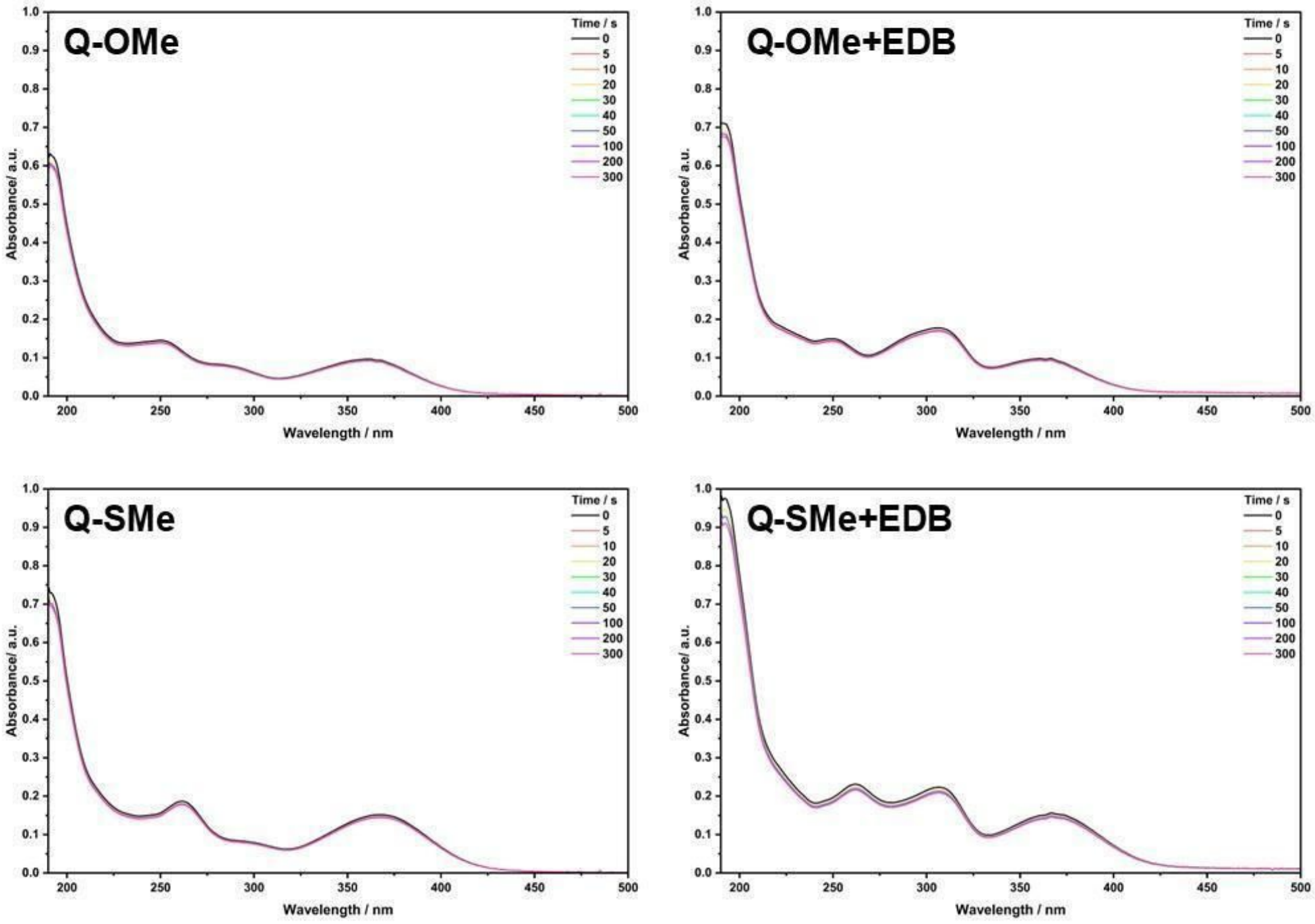

Figure S23. UV-Vis spectrum of EDB in acetonitrile.

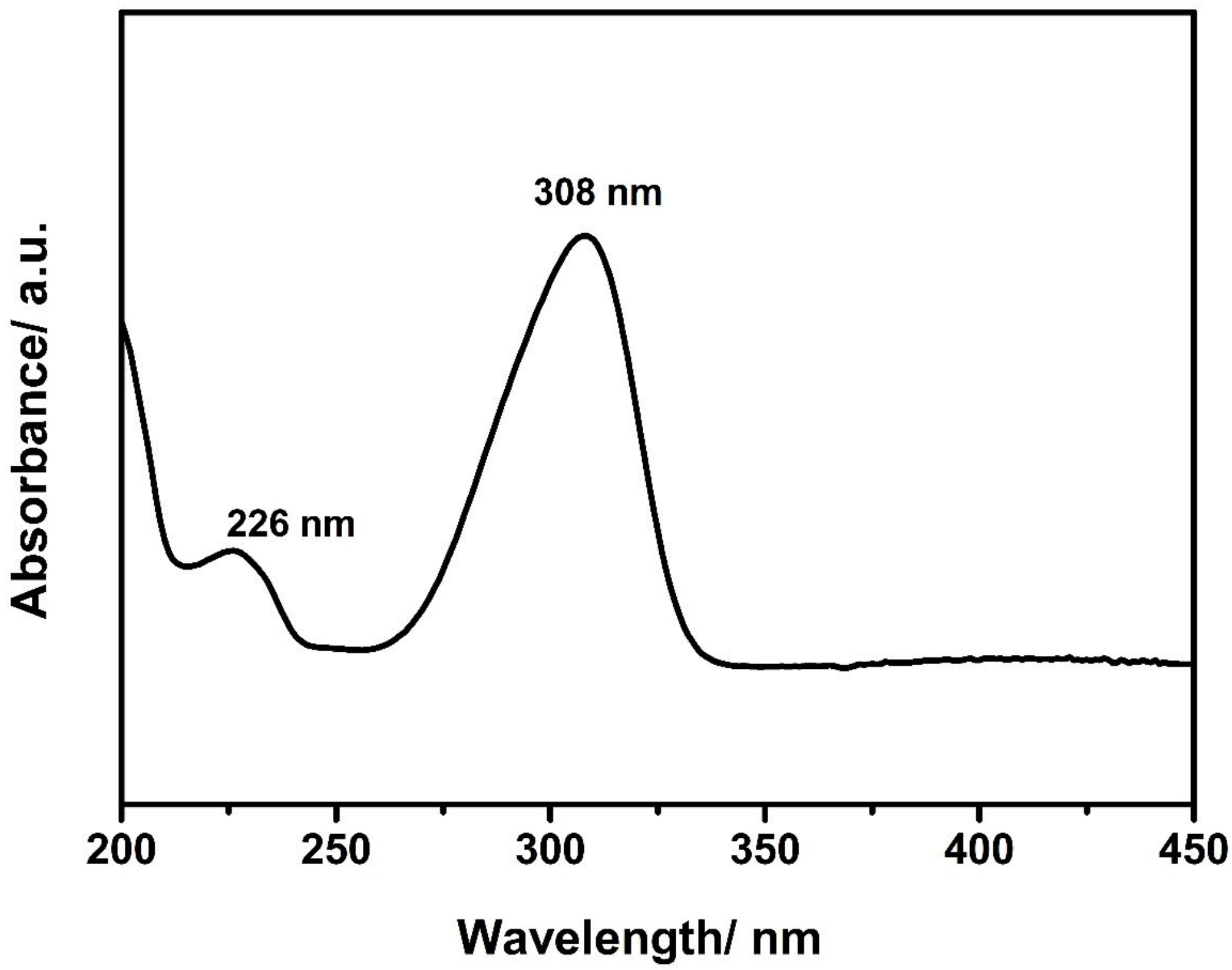

Figure S24. Mass spectrum of the final solution resulting from the photolysis of the **Q-SMe/EDB** system

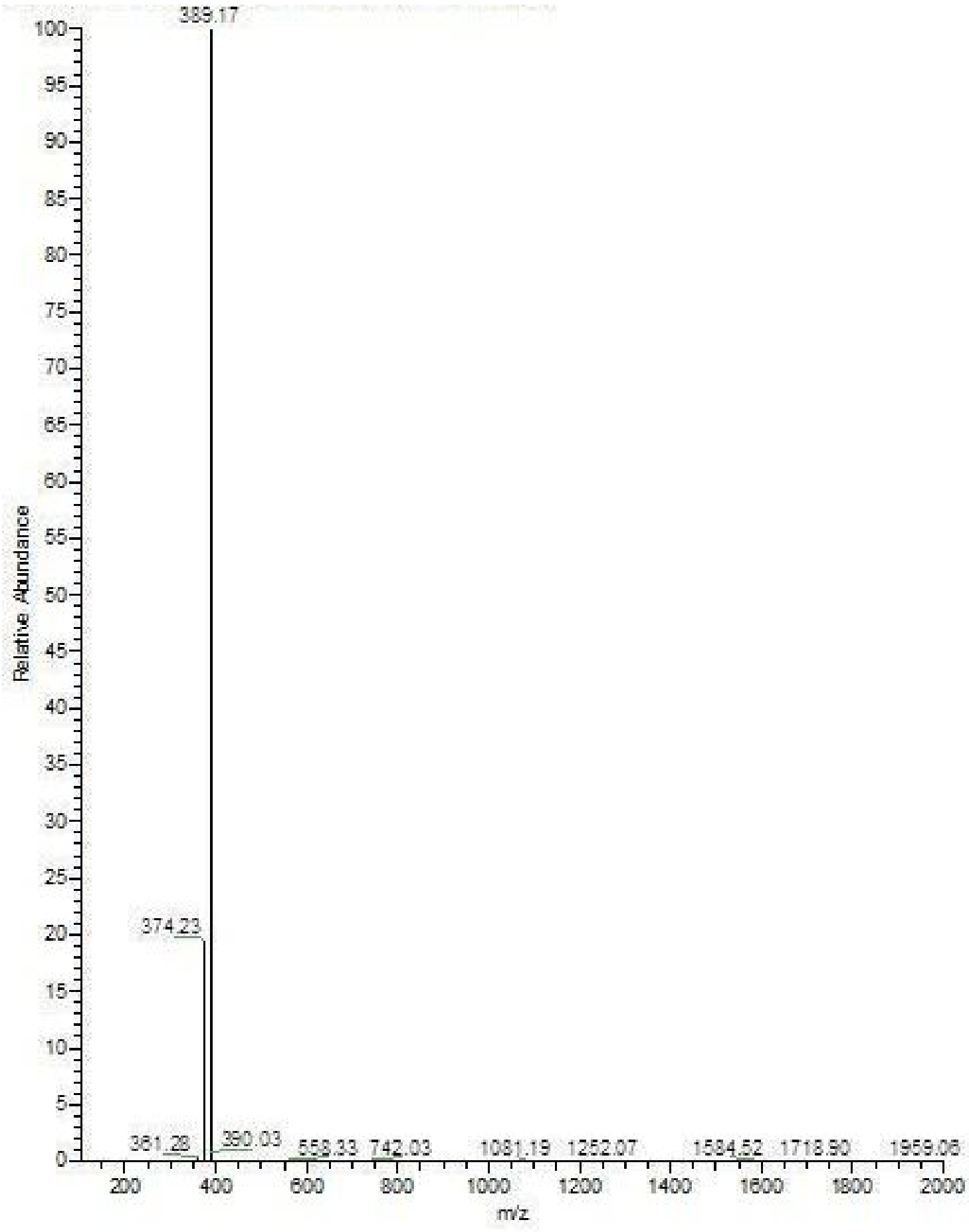

Figure S25. TG/DTG-DTA curves of polymers obtained from photopolymerization of formulations *a)* UDMA-I2, *b)* UDMA-I2-70, *c)* UDMA-I2-50, *d)* UDMA-I2-30, and *e)* UDMA-I2-0.

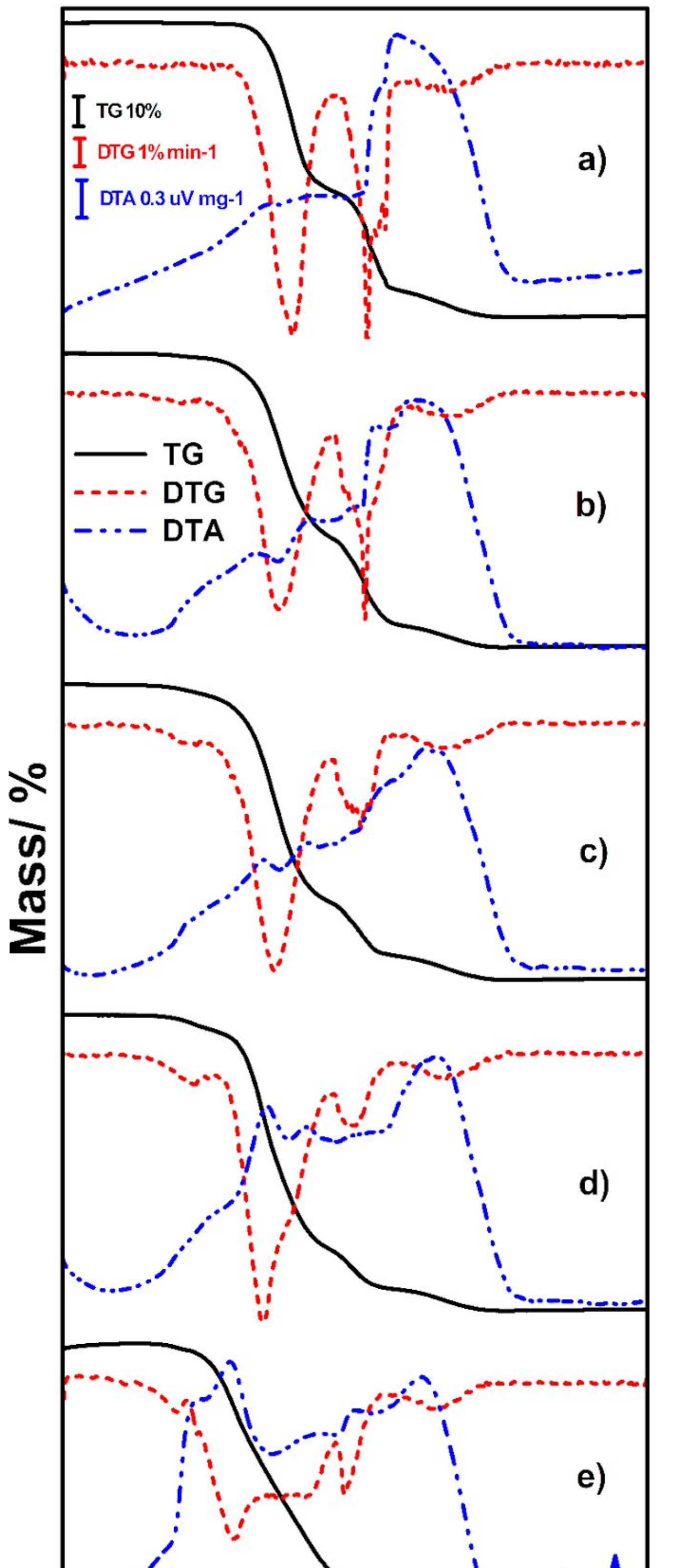

Figure S26. Objects obtained using the formulation UDMA-K2-70 in vat photopolymerization: Celtic flower (first line) under *a)* ambient light, *b)* and *c)* Micrographs at different regions. Honeycomb (second line): *d)* Superior view, *(f)* and *(g)* and *h)* Micrographs at different regions.

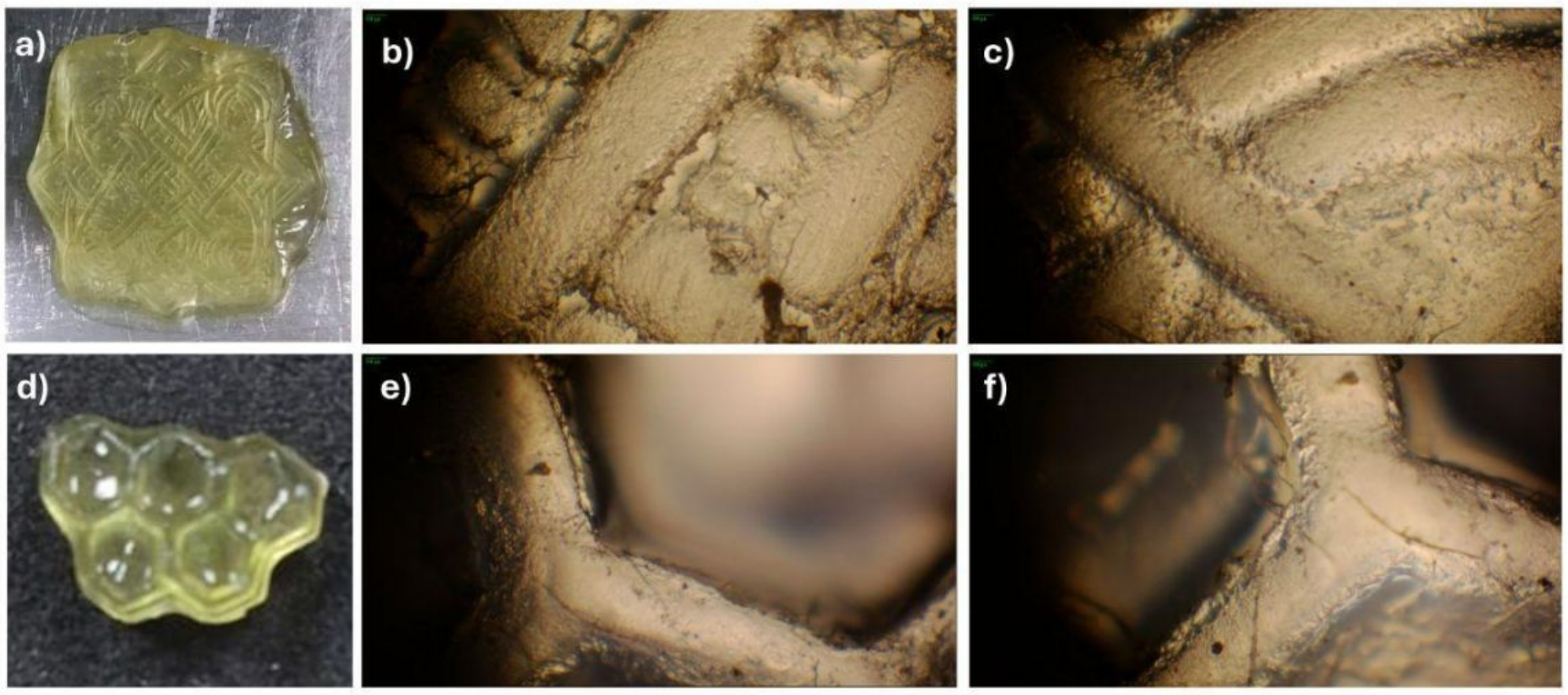

Figure S27. TG/DTG-DTA curves of polymer obtained from photopolymerization of formulation UDMA-K2-70.

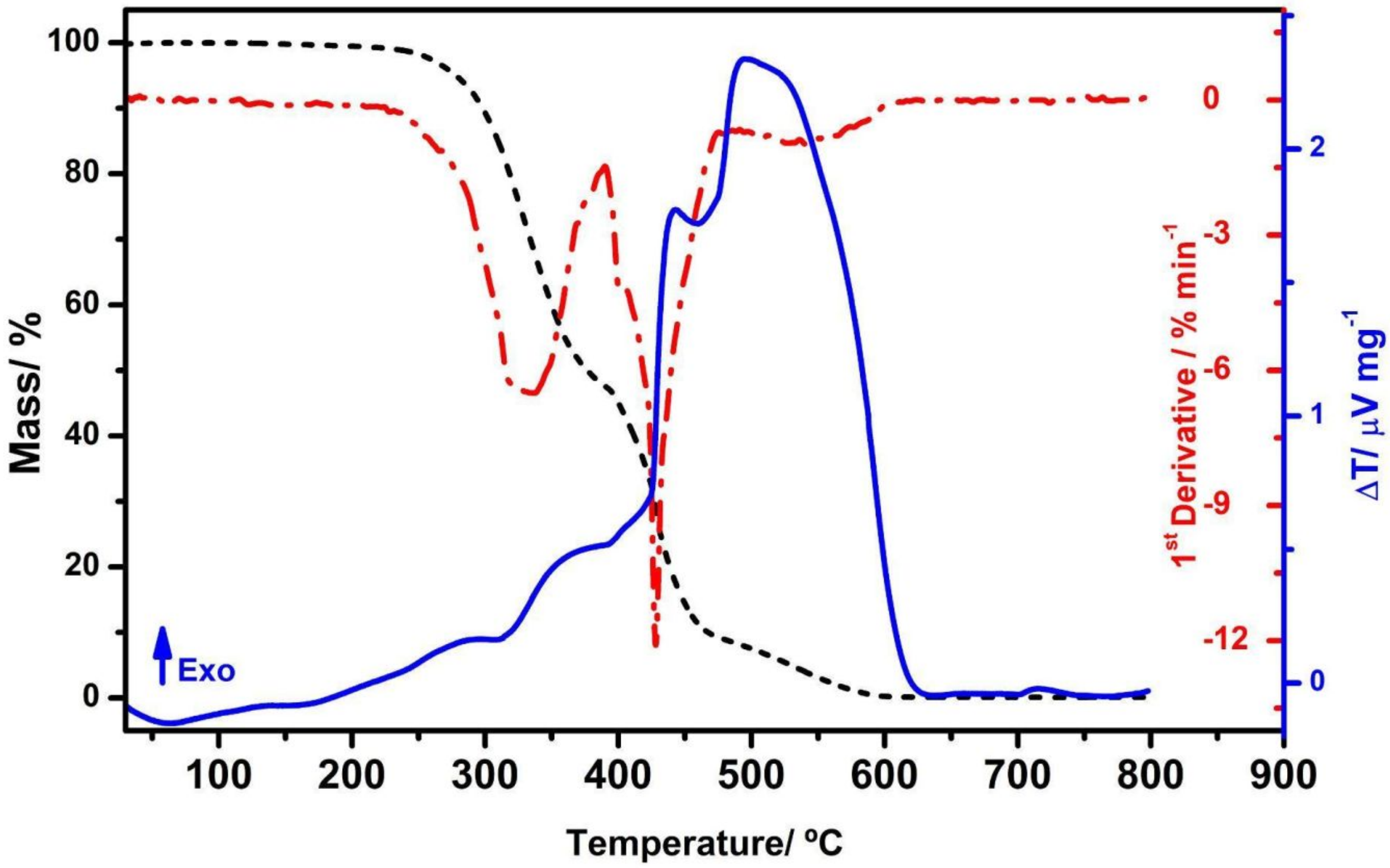

Table S1. Spectroscopic data of quinoline derivatives obtained by UV-Vis using acetonitrile as solvent in different concentrations.

**Quinoline photoinitiators**	**Maximum absorption/ nm**			
	**λ_1 (nm)**	**ε_1 (L mol^{-1}cm^{-1})**	**λ_2 (nm)**	**ε_2 (L mol^{-1} cm^{-1})**
**Q-OMe**	250	(1.99±0.03)x10^4	361	(1.87±0.02)x10^4
**Q-SMe**	262	(1.48±0.09)x10^4	368	(1.34±0.01)x10^4

Table S2. Temperature ranges (θ), mass losses(Δm), peak temperatures (T_P), maximum degradation rates (MDR), and temperature of maximum degradation rate (T_{MDR}) observed for each step in TG/DTG-DTA of quinoline derivatives.

**Sample**		**Step**			
		1st	*2nd*	*3rd*	*4th*
**Q-OMe**	θ/ ºC	270.0-410	410.0-668.0	-	-
	Δm / %	60.73	37.65	-	-
	T_p/ ºC	↑ 400.0	↑ 573.0	-	-
	T_{TMD}/ ºC	381.0	574.0	-	-
	MDR/ % min^{-1}	12.3	3.9	-	-
**Q-SMe**	θ/ ºC	180.0-214.0	213.4-357.0	357.0-490.0	490.0-702.0
	Δm / %	1.73	19.92	5.78	70.88
	T_p/ ºC	↓ 197.0	(304.0-366.0)*	-	↑ 571.0
	T_{TMD}/ ºC	196.0	335.0	-	633.0
	MDR/ % min^{-1}	1.3	3.6	-	6.0

Peak temperature; ↑ Exothermic peak; ↓ endothermic peak; *exotherm.

Table S3. Degree of Conversion (DC (%)) calculated by MIR for different systems of photopolymerization using UV and Blue lights (data are presented as mean ± standard deviation (n = 3).

Light	*System*	*DC (%)*					
		10s	*20s*	*50s*	*100s*	*200s*	*300s*
UV 370 nm	UDMA-A1	2.2±0.6	3.2±0.4	1.0±0.6	3.1±0.6	2.2±0.7	1.7±1.0
	UDMA-A2	0.7±0.6	0.6±0.6	0.7±0.7	1.2±0.7	1.4±1.0	1.3±1.1
	UDMA-B1	0.7±0.7	1.1±0.5	1.5±0.6	1.1±0.6	1.9±0.9	1.3±1.0
	UDMA-B2	0.7±0.5	1.0±0.4	1.0±0.4	1.4±0.4	1.8±1.0	1.7±1.1
Blue 456 nm	UDMA-A1	0.2±0.8	1.7±0.9	8.6±4.2	22.2±3.3	42.1±4.0	51.5±3.2
	UDMA-A2	0.5±0.5	1.0±1.6	1.5±1.4	10.1±4.9	33.2±4.1	48.3±3.7
	UDMA-B1	0.2±0.2	0.3±0.7	5.9±3.1	26.1±3.8	43.7±2.1	49.8±1.0
	UDMA-B2	8.7±3.0	29.9±3.3	50.1±2.8	60.5±3.2	68.2±3.1	73.3±3.4

Table S4. Rate of polymerization (Rp) calculated by MIR for different systems using blue light (456 nm)

System	*Rp (s^{-1})*					
	10s	*20s*	*50s*	*100s*	*200s*	*300s*
UDMA-A1	0.15	0.19	0.3	0.22	0.04	0.17
UDMA-A2	0.05	0.07	0.13	0.2	0.12	0.16
UDMA-B1	0.01	0.07	0.41	0.33	0.13	0.17
UDMA-B2	1.98	0.14	0.33	0.16	0.15	0.25

Table S5. Degree of conversion (DC (%)) calculated by MIR for different formulations containing different concentrations of PI or PIS under blue light. (data are presented as mean ± standard deviation (n = 3))

Quinoline Derivative	*System*	*DC (%)*					
		10s	*20s*	*50s*	*100s*	*200s*	*300s*
Q-OMe	UDMA-A1	0.2±0.8	1.7±0.9	8.6±4.2	22.2±3.3	42.1±4.0	51.5±3.2
	UDMA-A2	0.5±0.5	1.0±1.6	0.2±0.9	10.1±4.8	33.2±4.1	48.3±3.7
	UDMA-C1	0.8±0.3	2.2±1.3	13.2±7.7	28.4±6.7	39.4±4.6	45.3±2.4
	UDMA-C2	5.0±3.1	19.4±5.4	41.2±3.9	50.7±1.5	58.4±0.2	62.2±0.6
	UDMA-D1	0.9±1.7	0.4±2.3	8.8±1.8	26.3±1.0	40.6±0.7	48.1±1.0
	UDMA-D2	2.2±2.8	17.1±3.0	40.9±3.0	50.1±2.4	57.4±2.1	62.1±1.5
	UDMA-E1	0.1±0.8	1.1±0.9	2.8±0.6	16.7±2.0	31.0±1.8	37.6±1.4
	UDMA-E2	8.7±3.0	24.4±3.5	42.0±1.3	50.8±0.9	56.7±0.8	61.1±0.9
	UDMA-F1	0.6±0.5	0.1±0.3	0.3±0.5	14.0±3.3	24.4±1.6	30.0±1.5
	UDMA-F2	0.2±0.6	1.1±1.0	11.5±6.5	26.7±4.9	39.8±3.7	46.3±3.1
Q-SMe	UDMA-B1	0.2±0.2	0.3±0.7	5.9±3.1	26.1±3.8	43.7±2.1	49.8±1.0
	UDMA-B2	7.8±4.0	27.6±3.5	48.3±4.1	59.4±4.4	67.3±4.3	73.4±3.2
	UDMA-G1	0.3±0.3	9.6±2.1	36.3±3.5	48.1±2.0	56.8±2.1	62.0±2.7
	UDMA-G2	15.1±5.1	36.0±4.5	53.0±7.9	60.7±4.1	67.2±4.0	70.5±3.3
	UDMA-H1	1.8±0.6	7.8±0.4	19.1±1.6	27.2±0.8	36.2±1.8	42.6±2.7
	UDMA-H2	32.4±2.2	43.1±1.2	51.7±2.1	59.6±3.4	64.7±1.3	69.0±1.1
	UDMA-I1	5.6±2.1	16.3±3.1	33.3±4.3	43.9±4.0	51.7±4.0	56.2±4.2
	UDMA-I2	36.1±4.7	46.2±4.1	54.6±3.1	59.9±2.7	64.9±2.3	67.8±2.5
	UDMA-J1	3.1±0.9	10.4±1.2	22.7±0.9	30.4±0.7	41.6±1.2	49.2±1.3
	UDMA-J2	33.4±4.5	39.9±3.1	47.7±2.1	53.2±2.6	59.1±2.6	62.4±2.2

Table S6. Rate of polymerization (Rp (s^{-1})) calculated by MIR for different formulations containing different concentrations of PI or PIS under blue light.

Quinoline Derivative	***System***	***Rp (s^{-1})***					
		10s	***20s***	***50s***	***100s***	***200s***	***300s***
Q-OMe	UDMA-A1	0.15	0.19	0.30	0.22	0.04	0.17
	UDMA-A2	0.05	0.07	0.13	0.20	0.12	0.16
	UDMA-C1	0.14	0.31	0.49	0.26	0.06	0.15
	UDMA-C2	1.44	1.05	0.35	0.20	0.1	0.21
	UDMA-D1	0.05	0.06	0.42	0.20	0.01	0.16
	UDMA-D2	1.49	1.23	0.23	0.09	0.07	0.21
	UDMA-E1	0.10	0.04	0.22	0.20	0.02	0.12
	UDMA-E2	1.57	0.9	0.22	-0.01	0.12	0.20
	UDMA-F1	0.05	0.00	0.14	0.27	0.08	0.10
	UDMA-F2	0.09	0.26	0.47	0.12	0.04	0.15
Q-SMe	UDMA-B1	0.01	0.07	0.41	0.33	0.13	0.17
	UDMA-B2	1.98	0.14	0.33	0.16	0.15	0.25
	UDMA-G1	0.93	1.16	0.42	0.15	0.06	0.21
	UDMA-G2	2.09	0.95	0.21	0.05	0.09	0.24
	UDMA-H1	0.00	0.6	0.28	0.17	0.09	0.14
	UDMA-H2	1.07	0.34	0.14	0.14	0.02	0.23
	UDMA-I1	1.07	0.76	0.39	0.09	0.02	0.19
	UDMA-I2	1.01	0.59	0.15	0.10	0.08	0.23
	UDMA-J1	0.73	0.56	0.22	0.17	0.08	0.16
	UDMA-J2	0.65	0.38	0.13	0.05	0.04	0.21

Table S7. Temperature ranges (θ), mass losses(Δm), peak temperatures (T_P), maximum degradation rates (MDR), and temperature of maximum degradation rate (T_{MDR}) observed for each step in TG/DTG-DTA of polymers derived from formulations with different content of UDMA:TEGDMA.

Step		***UDMA-I2***	***UDMA-I2-70***	***UDMA-I2-50***	***UDMA-I2-30***	***UDMA-I2-0***	***UDMA-K2-70***
1st	*θ/ ºC*	250.0-380.0	176.0-215.0	172.0-217.0	165.0-235.0	153.0-195.0	238.0-391.0
	Δm / %	55.71	1.98	3.47	4.99	2.02	51.70
	T_p / ºC	(266.0-316.0)*	(176.0-238.0)*	(177.0-246.0)*	-	-	307.0 ↓ (321.0-392.0)*
	T_{TMD}/ ºC	333.0	182.0	-	205.0	182.0	336.8
	MDR/ % min^{-1}	9.9	0.4	-	1.1	1.0	6.5
2nd	*θ/ ºC*	380.0-482.0	215.0-388.0	217.0-381.0	235.0-378.0	195.0-388.0	391.0-475.4
	Δm / %	34.38	60.43	68.95	71.83	74.71	38.18
	T_p/ ºC	(430.0-454.0)*	(257.0-313.0)*	296.0 ↑ 355.0 ↑	300.0 ↑ 351.0 ↑	208.0 ↑ 251.0 ↑	443.0 ↑
	T_{MDR}/ ºC	431.0	317.0	310.0	294.0	254.0	428.0
	MDR/ % min^{-1}	10.1	7.9	9.0	9.7	5.6	12.1
3rd	*θ/ ºC*	482.0-603.0	388.0-480.0	381.0-465.0	378.0-471.0	388.0-451.0	475.4-603.5
	Δm / %	8.26	26.68	17.85	8.36	13.11	9.07
	T_p/ ºC	(454.0-632.0)*	(318.0-379.0)* (400.0-423.0)*	(423.0-471.0)*	(395.0-455.0)*	(395.0-453.0)*	503.0 ↑
	T_{MDR}/ ºC	539.0	428.0	424.0	407.0	401.0	540.0
	MDR/ % min^{-1}	1.1	8.2	3.8	2.7	4.0	1.0
4th	*θ/ ºC*	-	480.0-616.0	465.0-618.0	471.0-617.0	451.0-587.0	-
	Δm / %	-	7.15	8.14	7.11	7.31	-
	T_p/ ºC	-	(382.0-633.0)*	521.0 ↑	521.0 ↑	503.0 ↑	-
	T_{MDR}/ ºC	-	534.0	516.0	534.0	517.0	-
	MDR/ % min^{-1}	-	0.8	0.9	0.9	0.9	-
